# MVQTLCIM: composite interval mapping of multivariate traits in a hybrid F_1_ population of outbred species

**DOI:** 10.1186/s12859-017-1908-1

**Published:** 2017-11-23

**Authors:** Fenxiang Liu, Chunfa Tong, Shentong Tao, Jiyan Wu, Yuhua Chen, Dan Yao, Huogen Li, Jisen Shi

**Affiliations:** 1 The Southern Modern Forestry Collaborative Innovation Center, College of Forestry, Nanjing Forestry University, Nanjing, 210037 China; 2 College of Department of Computer Science and Engineering, Sanjiang University, Nanjing, 210012 China

**Keywords:** Quantitative trait locus, Composite interval mapping, Multivariate linear model, Multivariate traits, *Populus*

## Abstract

**Background:**

With the plummeting cost of the next-generation sequencing technologies, high-density genetic linkage maps could be constructed in a forest hybrid F_1_ population. However, based on such genetic maps, quantitative trait loci (QTL) mapping cannot be directly conducted with traditional statistical methods or tools because the linkage phase and segregation pattern of molecular markers are not always fixed as in inbred lines.

**Results:**

We implemented the traditional composite interval mapping (CIM) method to multivariate trait data in forest trees and developed the corresponding software, mvqtlcim. Our method not only incorporated the various segregations and linkage phases of molecular markers, but also applied Takeuchi’s information criterion (TIC) to discriminate the QTL segregation type among several possible alternatives. QTL mapping was performed in a hybrid F_1_ population of *Populus deltoides* and *P. simonii*, and 12 QTLs were detected for tree height over 6 time points. The software package allowed many options for parameters as well as parallel computing for permutation tests. The features of the software were demonstrated with the real data analysis and a large number of Monte Carlo simulations.

**Conclusions:**

We provided a powerful tool for QTL mapping of multiple or longitudinal traits in an outbred F_1_ population, in which the traditional software for QTL mapping cannot be used. This tool will facilitate studying of QTL mapping and thus will accelerate molecular breeding programs especially in forest trees. The tool package is freely available from https://github.com/tongchf /mvqtlcim.

**Electronic supplementary material:**

The online version of this article (10.1186/s12859-017-1908-1) contains supplementary material, which is available to authorized users.

## Background

Most forest trees are outbred species and have the characteristics of high heterozygosity and long generation times [1]. These properties make it very difficult to generate inbred lines in forest trees for linkage mapping and then for quantitative trait loci (QTL) mapping with traditional statistical methods. However, with the continuously reducing cost of next-generation sequencing (NGS) technologies and the development of new genetic mapping strategies, thousands of genetic markers could be obtained across many individuals and thus could be used to construct high-density genetic linkage maps in a forest hybrid F_1_ population [2, 3]. Such dense linkage maps would greatly facilitate QTL mapping as well as comparative genomics in forest trees. Yet, the statistical methods of QTL mapping used for populations derived from inbred lines cannot be directly applied to the outcrossing populations because the linkage phase and segregation pattern of markers on genetic maps may vary from locus to locus and are not always fixed as in inbred lines [4–6].

Over the past three decades, many statistical models developed for QTL mapping were mainly based on experimental populations, such as the backcross and F_2_, crossed from two inbred lines. These models initiated with the seminal approach of interval mapping (IM) proposed by Lander and Botstein [7]. To overcome the problem of possibly generating so-called ‘ghost QTL’ with IM [8], Zeng [9, 10] proposed the composite interval mapping (CIM) method by adding a proper number of background markers into the model to absorb effects of other QTLs outside the detected region. Since then, QTL mapping methods were extended to multiple interval mapping (MIM) [11, 12], and also to mapping binary and categorical traits [13, 14]. Moreover, Bayesian models [15–18] and the least absolute shrinkage and selection operator (LASSO) methods [19–23] were applied to mapping single or multiple QTLs. In addition, approaches were inherently established by extending from mapping single trait to multiple traits or longitudinal trait data [11, 24–26]. Specifically, Wu and colleagues proposed a so-called functional mapping method in order to identify QTLs that affect a particular biological process with trait values over multiple stages [27–30].

Meanwhile, several great efforts have been made to develop statistical models used for QTL mapping in outbred species. Haley et al. [31] proposed a method for identifying QTLs in an outcrossing population of pigs, but it has the limitations that it did not consider the possible changes in marker segregation pattern and the linkage phase of the parents. Besides the QTL location and effects, Lin et al. [32] subsequently established an approach that can estimate the linkage phase between the linked QTL and a marker in an outcrossed population. Tong et al. [6] proposed a model selection method to discriminate the most likely QTL segregation pattern within several possible QTL segregation patterns in a full-sib family generated from two outcrossing parents. This method actually was implemented in the context of IM, but it capitalized on the complex genetic architecture of an outcrossing population, such as the various marker segregation patterns and non-fixed linkage phases. Recently, Gazaffi et al. [33] presented a CIM method with a series of hypothesis tests to infer significant QTLs and their segregation types in a full-sib progeny. However, their procedure of testing significant QTLs is similar to the method in the software MapQTL [34], which could lose the power to detect QTLs segregating in the test cross or F_2_ pattern in real examples [6].

Although these significant advances have been achieved, there is still a lot of room to improve for QTL mapping in a hybrid F_1_ population in outbred species, because of the complex genetic characteristics of such a mapping population. In this study, we developed a model selection method to implement CIM for mapping tree height with values at different growth time points in a hybrid F_1_ population of *Populus deltoides* × *P. simonii*. The two *Populus* species display substantially different performance in growth rate, resistance to diseases and bad conditions, and rooting ability [2, 35]. Their hybrid progeny provide a permanent material for constructing genetic linkage maps and identifying QTLs in *Populus*. We showed that our QTL mapping approach can detect 12 QTLs that affect tree height, based on the parent-specific high-density linkage maps constructed in our previous study [2]. Furthermore, the new method can find more QTLs with higher significance compared with the interval mapping method. The software developed for implementing the algorithm can be downloaded from https://github.com/tongchf/mvqtlcim as package *mvqtlcim*.

## Methods

### Mapping population

The mapping population was an interspecific F_1_ bybrid population between *P. deltoides* (P_1_) and *P. simonii* (P_2_), which was established in 2011 [2]. The parental genetic linkage maps were constructed by using 1601 and 940 SNPs and covered 4249.12 and 3816.24 cM of the whole genomes of P_1_ and P_2_, respectively [2]. A total of 177 individuals were selected for QTL mapping. The tree height of each individual was measured at six different time points during the growth period in 2014. The phenotype data showed large variation at different development stages in the F_1_ hybrid population.

### Stepwise regression model

In order to apply CIM into an outbred full-sib family for multivariate phenotype data, the first step is to choose background markers to control other QTL effects when scanning a putative QTL at a specific position on genome. We used stepwise regression method to choose the background markers among the whole available molecular marker data. Considering *n* individuals with genotype data of *M* markers and the phenotypic values of a trait at *T* time points, the linear regression model can be described as

1 $$ {y}_{it}={\mu}_t+\sum \limits_{j=1}^M\sum \limits_{k=1}^{K_j}{x}_{ijk}{B}_{jkt}+{e}_{it},\kern1em i=1,\kern0.5em \cdots, \kern0.5em n;\kern1em t=1,\kern0.5em \cdots, \kern0.5em T $$

where *y*_*it*_ is the trait value at the *t*th time point of the *i*th individual; *μ*_*t*_ is the overall mean of the trait value at the *t*th time point; *x*_*ijk*_ is an indicator variable for the *k*th genotype of the *j*th marker for the *i*th individual, taking the value of 1 or 0; *B*_*jkt*_ is the effect of the *k*th genotype of the *j*th marker at the *t*th time point, with the restriction

2 $$ {\sum}_{k=1}^{K_j}{B}_{jkt}=0 $$

where *K*_*j*_ is the number of genotypes of the *j*th marker; *e*_*it*_ is a random error at the *t*th time point for the *i*th individual. Because a molecular marker could segregate in the ratio of 1:1, 1:2:1, or 1:1:1:1 in an outbred full-sib family [4, 5], the number of genotypes of the *j*th marker possibly takes the value of 2, 3, or 4. For the random errors of individual *i*, let ${e}_i^{\prime }=\left({e}_{i1},\kern0.5em {e}_{i2},\kern0.5em \cdots, \kern0.5em {e}_{iT}\right)$ and assume that *e*_*i*_~*N*(0,  *Σ*).

The stepwise regression involved starting with no markers in the model, adding a marker to the model with the most significance at a specified entry level, removing a candidate marker from the model if its significance is reduced below a specified staying level, and repeating this process until no markers can be added or deleted. Since model (1) actually belongs to a multivariate multiple regression model, the significance for a candidate marker in the model can be tested with Wilks’ lambda statistic

3 $$ \varLambda =\left|\widehat{\varSigma}\right|/\left|{\widehat{\varSigma}}_0\right| $$

where $\widehat{\varSigma}$ is the maximum likelihood estimate of *Σ* for the full model and ${\widehat{\varSigma}}_0$ for the reduced model under a null hypothesis. The lambda statistic can be approximated by an *F* or chi-square distribution in some cases for calculating *p*-value in testing the significance of a marker in our regression model [36, 37].

### Composite interval mapping model

Unlike in inbred lines, not only molecular markers but also QTLs may segregate in any patterns in an F_1_ outcrossing population. In our CIM model, we focused on the markers segregating in the types of *aa* × *ab*, *ab* × *aa*, *ab* × *ab* and *ab* × *cd*, and QTLs segregating in the types of test cross (i.e. *QQ* × *Qq* or *Qq* × *QQ*), F_2_ cross (i.e. *Qq* × *Qq* or *Qq* × *qQ*) and full cross (i.e. *Q*_1_ *Q*_2_ × *Q*_3_ *Q*_4_) [4, 6]. Assuming that there exists a QTL in an interval of markers M_s_ and M_s + 1_ on a chromosome, our CIM model for multivariate phenotype data can be described by incorporating the QTL genotype effects into model (1) as

4 $$ {y}_{it}=\sum \limits_{j=1}^J{x}_{ij}^{\ast }{\mu}_{jt}+\sum \limits_{\begin{array}{c}j=1\\ {}j\ne s,\kern0.5em s+1\end{array}}^M\sum \limits_{k=1}^{K_j}{x}_{ij k}{B}_{jkt}+{e}_{it},\kern1em i=1,\kern0.5em \cdots, \kern0.5em n;\kern0.5em t=1,\kern0.5em \cdots, \kern0.5em T $$

where *μ*_*jt*_ is the value of the *j*th QTL genotype at the *t*th time point; *J* is the number of QTL genotypes, determined by the QTL segregation type, possibly taking the value of 2, 3 or 4; *M* is the number of markers chosen as background markers in CIM; ${x}_{ij}^{\ast }$ is an indicator variable for the *j*th QTL genotype for the *i*th individual, taking the value of 1 or 0; The other variables are defined as in model (1). Let *B* denote the matrix composed of non-redundant parameters of *B*_*jkt*_ with the *t*th column corresponding to the *t*th time point, and *X*_*i*_ the row vector for individual *i* corresponding to the coefficients of parameter *B*_*jkt*_ s in any column of matrix *B*. The likelihood of the unknown parameters in model (2) can be written as

5 $$ L\left(\varTheta \right)=\prod \limits_{i=1}^n{L}_i\left(\varTheta \right)=\prod \limits_{i=1}^n\sum \limits_{j=1}^J{p}_{ij}f\left({y}_i;\kern0.5em {\mu}_j+{B^{\prime }{X}^{\prime}}_i,\kern0.5em \varSigma \right) $$

where *Θ* = (*μ*_1_,  ⋯,  *μ*_*J*_,  *B*,  *Σ*) is the unknown parameters,

$$ {\displaystyle \begin{array}{c}{y}_i={\left({y}_{i1}\kern0.5em {y}_{i2}\kern0.5em \cdots \kern0.5em {y}_{iT}\right)}^{\prime },\\ {}f\left({y}_i;\kern0.5em {\mu}_j+{B^{\prime }{X}^{\prime}}_i,\kern0.5em \varSigma \right)={\left(2\pi \right)}^{-\frac{T}{2}}{\left|\varSigma \right|}^{-\frac{1}{2}}{e}^{-\frac{1}{2}{\left({y}_i-{\mu}_j-{B^{\prime }{X}^{\prime}}_i\right)}^{\prime }{\varSigma}^{-1}\left({y}_i-{\mu}_j-{B^{\prime }{X}^{\prime}}_i\right)},\end{array}} $$

and *p*_*ij*_ is the conditional probability of the *j*th QTL genotype on the flanking marker genotype. Although there are many cases for the combination of any two markers due to several different marker segregation types, the conditional probability can be calculated in a uniform procedure [6].

Differentiating eq. (5) with respect to the unknown parameters of *μ*_*j*_s, *B* and *Σ*, and setting these partial derivatives to zero, we obtained the following equations as

6 $$ B={\left({X}^{\prime }X\right)}^{-1}{X}^{\prime}\left(Y-\sum \limits_{j=1}^J{P}_j\otimes {\mu}_j^{\prime}\right) $$

7 $$ {\mu}_j=\frac{\sum_{i=1}^n{P}_{ij}\left({y}_i-{B^{\prime }{X}^{\prime}}_i\right)}{\sum_{i=1}^n{P}_{ij}}\kern2.5em \left(j=1,\kern0.5em \cdots, \kern0.5em J\right) $$

8 $$ \varSigma =\frac{1}{n}\sum \limits_{i=1}^n\sum \limits_{j=1}^J{P}_{ij}\left({y}_i-{\mu}_j-{B^{\prime }{X}^{\prime}}_i\right){\left({y}_i-{\mu}_j-{B^{\prime }{X}^{\prime}}_i\right)}^{\prime } $$

Where

$$ Y=\left(\begin{array}{c}{y}_1^{\prime}\\ {}{y}_2^{\prime}\\ {}\vdots \\ {}{y}_n^{\prime}\end{array}\right),\kern0.5em X=\left(\begin{array}{c}{X}_1\\ {}{X}_2\\ {}\vdots \\ {}{X}_n\end{array}\right),\kern0.5em {P}_j=\left(\begin{array}{c}{P}_{1j}\\ {}{P}_{2j}\\ {}\vdots \\ {}{P}_{\mathrm{nj}}\end{array}\right)\kern1.5em \left(j=1,\kern0.5em \cdots, \kern0.5em J\right) $$

and

9 $$ {P}_{ij}=\frac{p_{ij}f\left({y}_i;\kern0.5em {\mu}_j+{B}^{\prime }{X}^{\prime },\kern0.5em \varSigma \right)}{\sum_{j=1}^J{p}_{ij}f\left({y}_i;\kern0.5em {\mu}_j+{B}^{\prime }{X}^{\prime },\kern0.5em \varSigma \right)}\kern2em \left(j=1,\kern0.5em \cdots, \kern0.5em J\right) $$

To obtain the maximum likelihood estimates (MLEs) of the unknown parameters, we performed the expectation-maximization (EM) algorithm [38]. In the E-step, the posterior probability of the *j*th QTL genotype for individual *i* was calculated by eq. (7) with initial values of the unknown parameters. In the M-step, the estimates of parameters *B*, *μ*_*j*_s and *Σ* were calculated by eqs. (4–6), respectively. The two steps were repeated until all the parameters converged.

To test if there is a significant QTL at a specified position of the genome, a null hypothesis was claimed as

10 $$ {H}_0:\kern0.5em {\mu}_1={\mu}_2=\cdots ={\mu}_J $$

The log-likelihood ratio (LR) statistic can be used for the test as

11 $$ LR=2\log \left[\frac{L\left(\widehat{\varTheta}\right)}{L\left({\widehat{\varTheta}}_0\right)}\right] $$

where $\widehat{\varTheta}$ and ${\widehat{\varTheta}}_0$ are the two MLE sets of all the parameters under the full model and the reduced model, respectively. Generally, the critical threshold for asserting a QTL existence can be determined by performing permutation tests [39, 40].

### QTL model selection

As described above, the QTL at a fixed position of the genome may segregate in several different patterns, but the true segregation pattern is unknown a priori. Therefore, a model selection method will be helpful to infer the QTL segregation. Here, we applied Akaike’s information criterion (AIC) [41], Bayesian information criterion (BIC) [42] and Takeuchi’s information criterion (TIC) [43] to infer the best QTL segregation pattern among the five alternatives. These criteria are defined as

12 $$ AIC=-2\log L\left(\widehat{\varTheta}\right)+2d $$

13 $$ BIC=-2\log L\left(\widehat{\varTheta}\right)+\log (n)d $$

14 $$ TIC=-2\log L\left(\widehat{\varTheta}\right)+2\mathrm{tr}\left(\widehat{J}\left(\widehat{\varTheta}\right){\widehat{I}}^{-1}\left(\widehat{\varTheta}\right)\right) $$

where $L\left(\widehat{\varTheta}\right)$ is the maximum likelihood of the model, *d* is the number of parameters to be estimated in the model, and $\widehat{J}\left(\widehat{\varTheta}\right)$ and $\widehat{I}\left(\widehat{\varTheta}\right)$ can be calculated as

15 $$ \widehat{J}\left(\widehat{\varTheta}\right)=\sum \limits_{i=1}^n\left(\frac{\partial \log {L}_i\left(\widehat{\varTheta}\right)}{\partial \varTheta}\right){\left(\frac{\partial \log {L}_i\left(\widehat{\varTheta}\right)}{\partial \varTheta}\right)}^{\prime } $$

16 $$ \widehat{I}\left(\widehat{\varTheta}\right)=-\frac{\partial^2\log L\left(\widehat{\varTheta}\right)}{\partial {\varTheta}^2} $$

The first and second derivatives involved in Eqs. (15) and (16) can be derived as in Additional file 1: Appendixes S1 and S2. We chose a proper index for discriminating QTL patterns by assessing the power of each criterion through computer simulations.

### Monte Carlo simulation

In order to validate the accuracy of parameter estimates and to evaluate the power of each model selection index, we performed a large number of computer simulations. Five chromosomes were considered in our simulations, each with 100 cM long and six markers evenly distributed. These simulated markers have the segregation types of *aa* × *ab*, *ab* × *aa*, *ab* × *ab* and *ab* × *cd*, and the linkage phases between any two adjacent markers are not fixed. Five QTLs with segregation patterns of *QQ* × *Qq*, *Qq* × *QQ*, *Qq* × *Qq*, *Qq* × *qQ* and *Q*_1_ *Q*_2_ × *Q*_3_ *Q*_4_ were supposed to control tree height in a growth period, whose positions on genome and genotype effects at eight sequential time points were set as shown in Tables 1 and Additional file 2: Tables S1-S5. Here, for consistency, a QTL genotype effect is defined as the deviation from the mean of the genotype values. The phenotype values of the *i*th individual were sampled from the multivariate normal distribution as *N*(*ν*_*i*_,  *Σ*), where the mean vector *ν*_*i*_ is the sum of the overall mean vector

$$ \nu =\left(34.46,\kern0.5em 69.94,\kern0.5em 83.93,\kern0.5em 103.54,\kern0.5em 114.73,\kern0.5em 120.80,\kern0.5em 124.01,\kern0.5em 125.69\right) $$

and the combination genotype effects of all the five QTLs involved over the eight time points. The covariance matrix *Σ* was determined by setting the sum of the heritabilities of all the five QTLs to 0.9 at each time point and the correlation coefficient of trait values between the *i*th and *j*th time points equal to 0.9^|*i* − *j*|^, which can be calculated as

17 $$ \varSigma =\left(\begin{array}{cccccccc}13.02& 18.38& 19.03& 20.97& 21.43& 20.81& 19.58& 18.08\\ {}18.38& 32.04& 33.17& 36.55& 37.34& 36.26& 34.12& 31.51\\ {}19.03& 33.17& 42.40& 46.72& 47.73& 46.36& 43.62& 40.28\\ {}20.97& 36.55& 46.72& 63.55& 64.92& 63.05& 59.33& 54.79\\ {}21.43& 37.34& 47.73& 64.92& 81.89& 79.53& 74.83& 69.10\\ {}20.81& 36.26& 46.36& 63.05& 79.53& 95.35& 89.73& 82.85\\ {}19.58& 34.12& 43.62& 59.33& 74.83& 89.73& 104.24& 96.25\\ {}18.08& 31.51& 40.28& 54.79& 69.10& 82.85& 96.25& 109.73\end{array}\right) $$

**Table 1:** The assumed QTL segregation patterns, positions on genome and the power of detecting the true QTL pattern with different model selection criteria under different sample sizes

Size	Pattern	Chromosome	Interval	Position	AIC	BIC	TIC
300	*QQ*×*Qq*	1	1	5	972	1000	975
	*Qq*×*Qq*	2	6	5	942	1000	947
	*Q*_*1*_ *Q*_*2*_×*Q*_*3*_ *Q*_*4*_	4	2	3	1000	920	1000
	*Qq*×*QQ*	4	5	8	984	1000	988
	*Qq*×*qQ*	5	3	7	918	1000	943
200	*QQ*×*Qq*	1	1	5	957	1000	960
	*Qq*×*Qq*	2	6	5	909	969	923
	*Q*_1_ *Q*_2_×*Q*_3_ *Q*_4_	4	2	3	998	634	991
	*Qq*×*QQ*	4	5	8	967	1000	972
	*Qq*×*qQ*	5	3	7	917	972	950
150	*QQ*×*Qq*	1	1	5	957	1000	950
	*Qq*×*Qq*	2	6	5	873	849	904
	*Q*_1_ *Q*_2_×*Q*_3_ *Q*_4_	4	2	3	986	426	925
	*Qq*×*QQ*	4	5	8	953	1000	928
	*Qq*×*qQ*	5	3	7	895	839	940

We considered sample sizes of 300, 200 and 150 each with 1000 replicates. For each case, the average parameter estimates and their standard deviations were calculated. In addition, under the three different model selection criteria described above, the power of detecting a specific QTL segregation pattern for each QTL model was obtained by counting the number of runs out of the 1000 repeats in which the correct pattern was chosen.

## Implementation

We developed a command-based software, namely *mvqtlcim*, to implement the computing for our CIM mapping method in an outbred full-sib family. *Mvqtlcim* was written in C++ with Boost C++ 1.62 (http://www.boost.org) and can run on Windows, Linux and Mac OS operating systems. The software utilizes a genetic linkage map constructed with different segregation molecular markers such as 1:1, 1:2:1 and 1:1:1:1, and assumes that QTL may segregate in the five different segregation patterns on a specific position of the genetic map. It allows users to select the best QTL segregation pattern with AIC, BIC and TIC for a significant QTL. It also provides command line parameters to be chosen for alternative analyses, including the number of background markers, window size [44], QTL segregation type, genetic map function and number of permutations. Specifically, when performing permutations to determine the empirical threshold of significant QTLs, *mvqtlcim* permits to use multithreads to accelerate computing speed. When an analysis completes, the software will generate two files for each QTL model, of which one contains the parameter estimates and the corresponding statistic values at every 1 cM on the genome, and the other saves the maximum LR value of each permutation. With these result files, we wrote an R script, *lrPlot.r*, to summarize the significant QTL information and generate scatter plots of LR against genome position. These plots can be optionally saved in pdf, jpg, png, tif or bmp format. The software and R script with the manuals are available from https://github.com/tongchf/mvqtlcim.

## Results

### Monte Carlo simulation

A large number of computer simulations were performed under different scenarios of sample sizes to assess the power of selecting the optimal QTL segregation pattern and the accuracy and precision of parameter estimates, using our multivariate CIM method with the background marker number of 5 and the window size of 15.0 cM. Table 1 shows the power of our statistical model to select the correct QTL segregation pattern among the five alternatives with AIC, BIC and TIC criteria under three different sample sizes. It is observed that all the powers for distinguishing the five QTL patterns are very high (≥93%) when the sample size is 300. Although the powers of BIC for the QTL segregation pattern of *Q*_1_ *Q*_2_ × *Q*_3_ *Q*_4_ are significantly lower (63.4% and 42.6%) under the sample sizes of 200 and 150, the powers of all the criteria for the other cases are still high (≥83.9%). It is interesting to note that the powers of AIC and TIC consistently keep high levels whatever the sample size is large or small, but the powers of TIC are more stable than those of AIC and keep at high levels of >90%. Therefore, the TIC criterion is highly recommended to use for selecting the best QTL segregation pattern with the CIM method developed here for an outbred F_1_ population.

Additional file 2: Tables S1-S5 list the parameter estimates in detail of the QTL position and genotype effects at each time point under the three cases of sample sizes. Overall, the estimated QTL positions tend towards the setting locations. But for the three QTL segregation patterns of Q_1_Q_2_ × Q_3_Q_4_, Qq × QQ and Qq × qQ, which were set in non-central locations, the position estimates are a little biased to the interval center. The average estimates of QTL genotype effects at the different time points for each case of the QTL segregation pattern are well close to the true values, but the standard deviations expand as expected when the sample size decreases from 300 to 150. Therefore, on average, the heritability of each QTL at each time point closes to the previously set value, and the sum of all the five QTL heritabilities at each time point is around the set value of 90% (Additional file 2: Table S6). In contrast, the estimate of the residual covariance matrix *Σ* for each QTL segregation pattern under each sample size expands averagely 2–3 times compared with the sum of the variances over the eight time points set in eq. (15) (Additional file 2: Table S7).

### QTL mapping in *Populus*

We performed QTL mapping for the tree heights over 6 time points in the F_1_ hybrid population of *P. deltoides* × *P. simonii* with the new developed tool *mvqtlcim*. The linkage maps used for QTL mapping were two parental specific maps; All the markers on the maternal map segregate in the type of *ab* × *aa*, while the markers on the paternal map in the type of *aa* × *ab* [2]. Therefore, the QTL segregation patterns were assumed to be *Qq* × *QQ* for the maternal map and *QQ* × *Qq* for the paternal map when scanning QTLs. In order to obtain the optimal mapping result, we ran *mvqtlcim* with different number of background markers and different window sizes, leaving the other optional parameters as defaults. The number of background markers was iterated from 3 to 39 with a step length of 2 and the window size from 5.0 to 30.0 cM with a step length of 5.0 cM. The optimal mapping result was defined as the one that all the significant QTLs account for the maximum proportion of the phenotypic variance in the population.

**Fig. 1:**
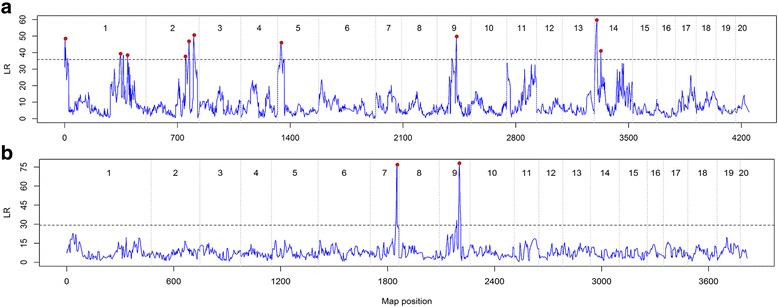
The profile of the log-likelihood ratios (LR) for detecting QTLs underlying tree height across the 20 linkage groups on each of the two parental genetic maps of (**a**) *P. deltoides* and (**b**) *P. simonii*. The threshold values for asserting the existence of a QTL at the significant level *p* = 0.05 are indicated as horizontal dashed lines that were determined by 1000 permutation tests. The vertical dashed lines separate the linkage groups. Each peak with a red dot is the highest one within a specified window size and represents a significant QTL

With the maternal linkage map, we found that the optimal mapping result corresponding to the run with 29 background markers and the window size of 20.0 cM, leading to 10 significant QTLs detected. The threshold determined by 1000 permutations was 35.84 for asserting the existence of a QTL at the significant level of 0.05. Fig. 1(a) displays the scatter plot of the LR against the position of the linkage map of *P. deltoides* with the dashed threshold line. A significant QTL corresponds to a peak which is above the threshold. If more than one significant peaks are within the specified window size, we chose the highest one as a significant QTL and ignored the others. It can be seen that the identified QTLs are distributed on the linkage groups of 1, 2, 5, 9 and 14. In the same way, we detected two significant QTLs on the paternal linkage map of *P. simonii* under the experiential threshold value of 29.23 with 3 background markers and the window size of 10.0 cM in running *mvqtlcim* (Fig. 1(b)). Table 2 summarizes the position, effects at each time point and the average heritability over the six time points for each significant QTL. These QTL IDs were named after the linkage group number, the order within a linkage group and either of the two parental linkage maps, where D stands for *P. deltoides* and S for *P. simonii* (e.g. Q2D1 indicates the second QTL located in group 1 on the linkage map of *P. deltoides*). It is observed that, on average, Q1D14 explains the maximum proportion (27.43%) of the phenotypic variance, while Q1D1 accounts for the minimum (only 1.11%).

**Table 2:** Summary of the identified QTL position, LR, effect of *QQ* at each time point and the average heritability over the time points on the two parental linkage maps of *P. deltoides* and *P. simonii*

Linkage Map	QTL ID	Chr/LG^a^	Marker Interval	Map Position (cM)	Genome Position^b^(Mb)	Region Length^b^(kb)	LR	T1^c^	T2	T3	T4	T5	T6	Average Heritability (%)
*P. deltoides*	Q1D1	1	2	2.12	1.24	701	48.24	−5.83	−4.39	−5.34	−7.32	−8.89	−11.59	1.11
	Q2D1	1	145	346.67	31.30	332	39.11	27.01	30.86	35.04	34.92	36.87	38.24	22.13
	Q3D1	1	159	390.19	35.01	57	38.11	−10.24	−13.18	−15.49	−18.51	−20.04	−24.91	6.00
	Q1D2	2	99	244.74	20.86	739	38.14	−26.04	−24.07	−22.74	−22.00	−21.80	−21.26	10.14
	Q2D2	2	102	265.38	19.82	1799	46.40	−29.44	−28.21	−27.24	−26.36	−26.57	−26.78	14.40
	Q3D2	2	111	296.05	23.72	222	49.57	23.82	25.13	23.93	22.51	23.07	23.25	10.67
	QD5	5	3	20.13	2.24	844	46.32	−17.64	−19.79	−21.67	−22.95	−22.22	−23.74	8.78
	QD9	9	44	119.31	8.18	344	49.43	21.82	26.66	24.76	27.05	25.34	21.66	11.60
	Q1D14	14	1	16.01	1.37	1734	59.85	−33.74	−37.42	−38.52	−40.54	−38.65	−38.13	27.43
	Q2D14	14	12	39.12	3.10	529	41.03	22.96	26.29	29.36	30.96	28.58	29.43	15.03
*P. simonii*	QS7	7	28	149.75	15.28	1115	77.47	7.16	7.90	12.36	11.03	19.11	29.92	5.25
	QS9	9	31	112.18	7.87	1200	77.59	11.91	14.76	19.68	19.57	25.60	35.70	9.74

^a^Chr, chromosome; LG, linkage group

^b^Estimated by the flanking SNPs on the reference sequence of *Populus trichocarpa* v3.0

^c^T1-T6 are the QTL effects of genotype QQ over six time point

### Candidate gene investigations

In order to investigate the candidate genes of these QTLs, we searched for the coding genes within the physical interval of each QTL in the gene annotation database of *Populus trichocarpa* v3.0 at Phytozome (https://phytozome.jgi.doe.gov). Because of the limited information in the annotation database [45], the coding sequences (CDS) of those genes related to each QTL were re-annotated by first blasting and then mapping on Gene Ontology (GO) terms with Blast2GO (https://www.blast2go.com). Consequently, the genomic region covering a QTL has an average length of 801 kb (Table 2) and contained 7–247 genes, of which 79% have 19.7 blast hits and 5.0 GO terms received on average (Additional file 3: Excel Sheets Q1D1-QS9). Additional file 4: Figures S1–12 showed the biological process GO category for the genes within the local region of each QTL. Interestingly, we found that the biological processes (BP) of three genes (Potri.014G029100, Potri.014G031100, Potri.014G031300) in the interval of Q1D14 and one gene (Potri.014G041600) in the interval of Q2D14 involved in brassinosteroids, which have great effects on plant height [46]. Another interesting finding was that two candidate genes (Potri.014G016500, Potri.014G027200) in the interval of Q1D14 and one gene in the interval of QD5 (Potri.005G021800) were related to shoot formation or development. Moreover, candidate genes for embryo or root development can be found in the flanking regions of Q1D14, QD5, QD9, QS7 and QS9, and for response to stress such as salt and heat in the regions of Q1D1, Q2D1, Q1D2, Q2D2, Q3D2, Q1D14 and QS9. Additionally, we also searched the candidate genes associated with photosynthesis, which plays the most important role in tree growth and development [47]. As a result, the candidate genes related to photosynthesis were located in the regions of Q1D2, Q2D2, Q1D14, QD5 and QS9. Other interesting candidate genes could be searched out in the Blast2GO annotation results presented in Additional file 3: Excel Sheets Q1D1-QS9 and Additional file 4: Figures S1–12.

## Discussion

Statistical methods for QTL mapping have been greatly developed for the past three decades, from the seminal work of interval mapping by Lander and Botstein [7] to the recent more popular Bayesian LASSO approaches [19–23]. However, there were few successful examples of identifying QTLs in outbred forest trees. One of the reasons may be due to the fact that most QTL mapping tools are not for the outbred species in which inbred lines are difficultly or even impossibly derived. Here, we implemented the traditional CIM method into an F_1_ population generated by hybridizing two outbred parents for mapping multiple or longitudinal traits. It is essentially useful for forest trees because such species have the characteristic of long generation times and high heterozygosity so that phenotypic data over long time can be easily observed but the genetic structures are more complicated. With the model selection criterion of TIC, our method could discriminate a QTL segregation type among five alternatives with a higher power (see section 3.2). In contrast to our previous work [6], the IM method with the LEC criterion for mapping a single trait could select an appropriate QTL segregation type by considering only three alternative patterns. Compared with the recent work of Gazaffi et al. [33], our work has a great advantage in the aspect of inferring a QTL segregation pattern (as described in introduction). We also provided the software *mvqtlcim* to put our method into practice. The software permits to use multithreads for performing a large number of permutations to determine the experimental threshold of LR for a significant QTL.

With the multivariate linear model method and EM algorithm, our CIM approach for mapping multivariate data traits has the advantage that the MLEs of unknown parameters can be globally obtained with limited iterative steps at each position on genome. However, in most functional QTL mapping cases [27, 28, 48, 49], owing to the nonlinear growth curves involved in the statistical models, the parameter MLEs could not be always obtained globally. This may decrease the power of identifying QTLs or even possibly generate pseudo QTLs. To overcome the problem in functional QTL mapping, we could first use the multivariate CIM method proposed here to identify QTLs and then to find the growth curves of these QTL genotypes. One strategy is to derive the nonlinear growth curve using the function mapping method within a small region flanking a QTL, which allows to obtain the optimal solution by iterating over different initiative points with intensive computing. Another way is to directly fit the growth curve with the QTL genotype values over time estimated from our multivariate CIM method. The latter method was illustrated by fitting the estimated genotype values for the 12 QTLs identified in section 3.3 with the Richards’ growth curves [50] (Additional file 5: Figure S13).

The results of Monte Carlo simulations indicated that our QTL mapping approach can provide accurate estimates of genetic parameters and a high power of inferring the actual QTL segregation type, but the estimate of the residual covariance matrix *Σ* expanded several times (Additional file 2: Table S7) compared with the setting values. It is noted that the estimate of residual variance was not assessed and ignored in the pioneer work of CIM approach [10]. This inconsistency between the estimates and the setting values in the residual covariance matrix could be explained by the fact that the setting model for simulations contains all the five QTL effects while the CIM model focuses only one QTL effect at a specific position on genome. The other QTL effects cannot be fully absorbed by the background markers in the CIM model, thus leading to the expanded estimates of the residual errors.

The application of mapping QTLs in *Populus* illustrated that our new multivariate CIM method could detected more number of QTLs underlying tree height in this study than in a previous study (12 vs. 8), in which a modified CIM was applied for tree height measured at a single time point [2]. These included some small-effect QTLs, such as Q1D1, QS9 and Q3D1 that averagely accounted for 1.11%, 5.25% and 6.00% of the phenotypic variances over the 6 time points, respectively (Table 2). This may be the main reason that our QTL mapping approach allows more QTLs to be detected. We also noted that the QTL effect size was not consistent with the LR statistic in our multivariate CIM mapping. For example, Q1D1 has a bigger LR value than Q2D2 (48.24 vs. 39.11), but its heritability is much lower than the later (1.11% vs. 22.13%) (Table 2). The reason is that the CIM statistical model may be different for different positions on genome because the background markers and their number in the model vary with the detected position. Therefore, the LR values of QTLs cannot be compared with each other to determine if one would more significant than the other. However, the LR threshold for significant QTLs is strictly valid in statistics because it was determined by the LR values each with the largest value chosen from a different permutation over the whole genome positions.

Further compared with other previous studies in mapping *Populus* height, our method may not only find more number of QTLs but also increase the genetic variance explained by them. In the early 1990s, Bradshaw and Stettler [51] found one QTL underlying 2-year height on linkage group D, which accounted for 25.9% of the phenotypic variance in an F_2_ population derived by crossing *P. trichocarpa* and *P. deltoides*. Later on, Wu (1998) [52] detected two QTLs for 3-year height on linkage groups D and M with the same materials, totally explaining 27.3% of the phenotypic variance. Because the relationship between *Populus* linkage groups and chromosomes was not so clear in the early two studies, we could not match the QTL positions to our present results. Recently, Monclus et al. (2012) [45] identified 5 QTLs distributed on chromosomes 1, 5, 6, 10 and 14 for the first-year height (Height1) and 7 QTLs on chromosomes 4, 6, 10, 12, 13, 16 and 17 for the second-year height (Height2) using 330 F_1_ *P. deltoides* × *P. trichocarpa* progeny. These QTLs could explain 20~30% of the phenotypic variance for Height1 or Height2, but only two QTLs were located consistently on the same chromosomes (6 and10) for the heights of the 2 years even with the same mapping materials. Among these QTLs, three for Height1 estimated in the confidence intervals of 23.12–54.23 Mb on chromosome 1, 7.11–25.80 Mb on chromosome 5, and 4.87–12.49 Mb on chromosome 14 seem to be in agreement with the QTLs identified in this study that were located in the positions of 31.30/35.01 Mb (Q2D1/Q3D1), 2.24 Mb (QD5), and 1.37 Mb (Q1D14) on the corresponding chromosomes. More recently, Du et al. [53] identified three QTLs affecting tree height in an F_1_ population of *Populus*, which were located in linkage groups 8 (Chr01), 12 and 16 (Chr13), and accounted for 3.4%, 8.0% and 6.4% of the phenotypic variance, respectively. One QTL was estimated in the interval of 18.37–21.00 Mb on the same chromosome (Chr01) as the QTLs of Q1D1, Q2D1 and Q3D1 detected in this study, but it was over 10 Mb away from any one of the three QTLs (Table 2). These comparisons between the previous and current studies displayed a large difference in identifying QTLs for *Populus* height, though a few consistent cases existed. The reason may be due to many factors such as mapping materials, genetic data structures, measures of phenotypic traits, and statistical methods [54].

Finally, we also conducted QTL analysis for our *Populus* real datasets each from one parental linkage map using the popular LASSO method with the glmnet/R package (v2.0–10, http://www.stanford.edu/~hastie/Papers/glmnet.pdf). In order to select a stable optimal value of the tuning parameter, the leave-one-out cross-validation was performed for each dataset (Additional file 6: Figure S14). As a result, a total of 12 SNPs were identified to be associated with the tree height, exactly half of which come from each SNP dataset. Among these associated SNPs, three were detected consistently by both CIM and LASSO (Additional file 6: Table S8). The high level of inconsistency between the results of CIM and LASSO was also observed in the most recent work of Xu and his colleagues [54], where they identified 28 and 29 QTLs for eight yield traits in maize by CIM and LASSO, respectively, but only half were consistent with both methods. The reason may be due to the difference in the way to utilize marker information in the two methods. The CIM method takes use of not only the marker segregation information but also the information of marker linkage as well as linkage phase, thus capable of detecting a QTL in an interval of two adjacent markers. However, although LASSO can handle a whole marker dataset simultaneously, it only uses the marker genotype information and provides the associated information between markers and a phenotypic trait. Perhaps, each of the two methods has its own advantages in such a hard task of QTL identification.

## Conclusion

The traditional CIM method was implemented for mapping multiple or longitudinal traits in a full-sib family derived by crossing two outbred parents. Our method not only incorporated various marker segregation ratios, such as 1:1, 1:2:1 and 1:1:1:1, but also utilized the model selection index of TIC to discriminate the actual QTL segregation pattern among several possible alternatives. We provided a powerful tool package to implement the algorithms of our method, which is freely available at the website: https://github.com/tongchf/mvqtlcim. The software package will facilitate studying of QTL mapping and thus will accelerate molecular breeding programs especially in forest trees.

## Additional files

Additional file 1: Appendices S1 and S2. (DOCX 93 kb)

Additional file 2: Table S1. Average of parameter estimates with the standard deviation in bracket under different sample sizes when the QTL segregation type is *QQ* × *Qq*, based on 1000 simulation replicates. **Table S2.** Average of parameter estimates with the standard deviation in bracket under different sample sizes when the QTL segregation type is *Qq* × *Qq*, based on 1000 simulation replicates. **Table S3.** Average of parameter estimates with the standard deviation in bracket under different sample sizes when the QTL segregation type is *Q1Q2* × *Q3Q4*, based on 1000 simulation replicates. **Table S4.** Average of parameter estimates with the standard deviation in bracket under different sample sizes when the QTL segregation type is *Qq* × *QQ*, based on 1000 simulation replicates. **Table S5.** Average of parameter estimates with the standard deviation in bracket under different sample sizes when the QTL segregation type is *Qq* × *qQ*, based on 1000 simulation replicates. **Table S6.** Summary on average estimates of QTL heritabilities (%) with the standard deviation in brackets under different time points (T1-T8) and different sample sizes based on 1000 simulation replicates. **Table S7.** The average estimate of the residual covariance matrix with standard deviations in brackets when the sample size is 300 and the QTL segregation type is *Qq* × *qQ*, based on 1000 simulation replicates. (DOCX 42 kb)

Additional file 3: Excel Sheets from Q1D1 to QS9. (XLSX 97 kb)

Additional file 4: Figure S1. Biological process GO category for the genes within the region of QTL Q1D1. **Figure S2.** Biological process GO category for the genes within the region of QTL Q2D1. **Figure S3.** Biological process GO category for the genes within the region of QTL Q3D1. **Figure S4.** Biological process GO category for the genes within the region of QTL Q1D2. **Figure S5.** Biological process GO category for the genes within the region of QTL Q2D2. **Figure S6.** Biological process GO category for the genes within the region of QTL Q3D2. **Figure S7.** Biological process GO category for the genes within the region of QTL QD5. **Figure S8.** Biological process GO category for the genes within the region of QTL QD9. **Figure S9.** Biological process GO category for the genes within the region of QTL Q1D14. **Figure S10.** Biological process GO category for the genes within the region of QTL Q2D14. **Figure S11.** Biological process GO category for the genes within the region of QTL QS7. **Figure S12.** Biological process GO category for the genes within the region of QTL QS9. (DOCX 642 kb)

Additional file 5: Figure S13. Richards’ growth curves of the 12 QTLs underlying the tree height of *Populus*, fitted with their genotype values (dot) over time estimated from the multivariate CIM method. The red is for the genotype *QQ* and the blue for *Qq*. (PDF 375 kb)

Additional file 6: Figure S14. Plots of the mean cross-validated error against the log of parameter lambda for the female (a) and male (b) SNP datasets. **Table S8.** SNPs identified to be associated with *Populus* height by the LASSO method using the two SNP datasets from each parental linkage map. (DOCX 61 kb)

## Abbreviations

AIC: Akaike’s information criterion
BIC: Bayesian information criterion
CIM: Composite interval mapping
EM: Expectation-maximization
IM: Interval mapping
LASSO: Least absolute shrinkage and selection operator
LR: Log-likelihood ratio
MLE: Maximum likelihood estimate
NGS: Next-generation sequencing
QTL: Quantitative trait locus
TIC: Takeuchi’s information criterion
