# Supplementary material for: MVQTLCIM: composite interval mapping of multivariate traits in a hybrid F1 population of outbred species
Source: BMC Bioinformatics. 2017 Nov 23;18:515. doi: 10.1186/s12859-017-1908-1 (PMC5701343; doi:10.1186/s12859-017-1908-1)
Supplement: Supplementary file 1 — (DOCX 93 kb) [file 12859_2017_1908_MOESM1_ESM.docx]

**Appendix S1.**

To calculate$\hat{J}(\hat{\Theta})$, the first derivatives of $L_{i}\left(\Theta\right)=\sum_{j=1}^{J} p_{ij}f(y_{i}; \mu_{j}+B'X'_{i}, \Sigma)$ with respect to the unknown parameters can be expressed as

$$\frac{\partial\ln L_{i}}{\partial\mu_{j}}=P_{ij}\Sigma^{-1}(y_{i}-\mu_{j}-B'X'_{i})$$

$$\frac{\partial\ln L_{i}}{\partial B}=\sum_{j=1}^{J} P_{ij}X'_{i}(y_{i}-\mu_{j}-B'X'_{i})'\Sigma^{-1}$$

$$\begin{matrix} \frac{\partial\ln L_{i}}{\partial\Sigma}=-\frac{1}{2}\frac{\partial\ln\left|\Sigma\right|}{\partial\Sigma}+\sum_{j=1}^{J} P_{ij}\frac{\partial}{\partial\Sigma}\left[-\frac{1}{2}(y_{i}-\mu_{j}-B'X'_{i})'\Sigma^{-1}(y_{i}-\mu_{j}-B'X'_{i})\right] \\ \text{=}-\Sigma^{-1}+\frac{1}{2}\mathrm{diag}(\Sigma^{-1})+\Sigma^{-1}\left[\sum_{j=1}^{J} P_{ij}(y_{i}-\mu_{j}-B'X'_{i})(y_{i}-\mu_{j}-B'X'_{i})'\right]\Sigma^{-1} \\ -\frac{1}{2}\mathrm{diag}\left\{\Sigma^{-1}\left[\sum_{j=1}^{J} P_{ij}(y_{i}-\mu_{j}-B'X'_{i})(y_{i}-\mu_{j}-B'X'_{i})'\right]\Sigma^{-1}\right\} \end{matrix}$$

where

$$P_{ij}=\frac{p_{ij}e^{-\frac{1}{2}(y_{i}-\mu_{j}-B'X'_{i})'\Sigma^{-1}(y_{i}-\mu_{j}-B'X'_{i})}}{\sum_{j'=1}^{J} p_{ij'}e^{-\frac{1}{2}(y_{i}-\mu_{j'}-B'X'_{i})'\Sigma^{-1}(y_{i}-\mu_{j'}-B'X'_{i})}}$$

**Appendix S2.**

In order to computer$\hat{I}(\hat{\Theta})$, we first express the first derivatives of $\ln L$ as following

$$\begin{matrix} \frac{\partial\ln L}{\partial\mu_{j}}=\sum_{i=1}^{n} P_{ij}\frac{\partial}{\partial\mu_{j}}\left[-\frac{1}{2}(y_{i}-\mu_{j}-B'X'_{i})'\Sigma^{-1}(y_{i}-\mu_{j}-B'X'_{i})\right] \\ =\sum_{i=1}^{n} P_{ij}\frac{\partial}{\partial\mu_{j}}\left[-\frac{1}{2}(y_{i}-B'X'_{i})'\Sigma^{-1}(y_{i}-B'X'_{i})+(y_{i}-B'X'_{i})'\Sigma^{-1}\mu_{j}-\frac{1}{2}\mu'_{j}\Sigma^{-1}\mu_{j}\right] \\ =\Sigma^{-1}\sum_{i=1}^{n} P_{ij}(y_{i}-\mu_{j}-B'X'_{i}) \end{matrix}$$

$$\begin{matrix} \frac{\partial\ln L}{\partial B}=\sum_{i=1}^{n} \sum_{j=1}^{J} P_{ij}\frac{\partial}{\partial B}\left[-\frac{1}{2}(y_{i}-\mu_{j}-B'X'_{i})'\Sigma^{-1}(y_{i}-\mu_{j}-B'X'_{i})\right] \\ =\sum_{i=1}^{n} \sum_{j=1}^{J} P_{ij}\frac{\partial}{\partial B}\mathrm{tr}\left[-\frac{1}{2}\Sigma^{-1}(y_{i}-\mu_{j}-B'X'_{i})(y_{i}-\mu_{j}-B'X'_{i})'\right] \\ =\sum_{i=1}^{n} \sum_{j=1}^{J} P_{ij}\frac{\partial}{\partial B}\mathrm{tr}\Sigma^{-1}\left[-\frac{1}{2}(y_{i}-\mu_{j})(y_{i}-\mu_{j})'+(y_{i}-\mu_{j})X_{i}B-\frac{1}{2}B'X'_{i}X_{i}B\right] \\ =\sum_{i=1}^{n} \sum_{j=1}^{J} P_{ij}\left[X'_{i}(y_{i}-\mu_{j})'\Sigma^{-1}-X'_{i}X_{i}B\Sigma^{-1}\right] \\ =\left[X'\left(Y-\sum_{j=1}^{J} P_{j}\otimes\mu'_{j}\right)-X'XB\right]\Sigma^{-1} \end{matrix}$$

$$\begin{matrix} \frac{\partial\ln L}{\partial\Sigma}=-\frac{n}{2}\frac{\partial\ln\left|\Sigma\right|}{\partial\Sigma}+\sum_{i=1}^{n} \sum_{j=1}^{J} P_{ij}\frac{\partial}{\partial\Sigma}\left[-\frac{1}{2}(y_{i}-\mu_{j}-B'X'_{i})'\Sigma^{-1}(y_{i}-\mu_{j}-B'X'_{i})\right] \\ \text{=}-n\Sigma^{-1}+\frac{n}{2}\mathrm{diag}(\Sigma^{-1})+\sum_{i=1}^{n} \sum_{j=1}^{J} P_{ij}\left[\Sigma^{-1}(y_{i}-\mu_{j}-B'X'_{i})(y_{i}-\mu_{j}-B'X'_{i})'\Sigma^{-1}\right. \\ \left. -\frac{1}{2}\mathrm{diag}\Sigma^{-1}(y_{i}-\mu_{j}-B'X'_{i})(y_{i}-\mu_{j}-B'X'_{i})'\Sigma^{-1}\right] \\ =-n\Sigma^{-1}+\frac{n}{2}\mathrm{diag}(\Sigma^{-1})+\Sigma^{-1}\left[\sum_{i=1}^{n} \sum_{j=1}^{J} P_{ij}(y_{i}-\mu_{j}-B'X'_{i})(y_{i}-\mu_{j}-B'X'_{i})'\right]\Sigma^{-1} \\ -\frac{1}{2}\mathrm{diag}\left\{\Sigma^{-1}\left[\sum_{i=1}^{n} \sum_{j=1}^{J} P_{ij}(y_{i}-\mu_{j}-B'X'_{i})(y_{i}-\mu_{j}-B'X'_{i})'\right]\Sigma^{-1}\right\} \end{matrix}$$

and then the second derivatives can be calculated as

$$\begin{matrix} \frac{\partial}{\partial\mu_{k}}\left(\frac{\partial\ln L}{\partial\mu_{j}}\right)=\Sigma^{-1}\sum_{i=1}^{n} \frac{\partial}{\partial\mu_{k}}\left(P_{ij}(y_{i}-\mu_{j}-B'X'_{i})\right) \\ =\Sigma^{-1}\sum_{i=1}^{n} \left[\left(y_{i}-\mu_{j}-B'X'_{i}\right)\left(\frac{\partial P_{ij}}{\partial\mu_{k}}\right)^{'}+P_{ij}\frac{\partial(y_{i}-\mu_{j}-B'X'_{i})}{\partial\mu_{k}}\right] \\ =\left\{\begin{matrix} \Sigma^{-1}\sum_{i=1}^{n} \left(y_{i}-\mu_{j}-B'X'_{i}\right)\left(\frac{\partial P_{ij}}{\partial\mu_{k}}\right)^{'} k\neq j \\ \Sigma^{-1}\sum_{i=1}^{n} \left[\left(y_{i}-\mu_{j}-B'X'_{i}\right)\left(\frac{\partial P_{ij}}{\partial\mu_{k}}\right)^{'}-P_{ij}I\right] k=j \end{matrix}\right. \end{matrix}$$

$$=\left\{\begin{matrix} -\Sigma^{-1}\left[\sum_{i=1}^{n} P_{ij}P_{ik}\left(y_{i}-\mu_{j}-B'X'_{i}\right)\left(y_{i}-\mu_{k}-B'X'_{i}\right)^{'}\right] \Sigma^{-1} k\neq j \\ \Sigma^{-1}\left[\sum_{i=1}^{n} P_{ij}(1-P_{ij})\left(y_{i}-\mu_{j}-B'X'_{i}\right)\left(y_{i}-\mu_{j}-B'X'_{i}\right)^{'}\right] \Sigma^{-1}-\left(\sum_{i=1}^{n} P_{ij}\right) \Sigma^{-1} k=j \end{matrix}\right.$$

where $\frac{\partial P_{ij}}{\partial\mu_{k}}=\left\{\begin{matrix} (1-P_{ij})P_{ij}\Sigma^{-1}(y_{i}-\mu_{j}-B'X'_{i}) k=j \\ -P_{ij}P_{ik}\Sigma^{-1}(y_{i}-\mu_{k}-B'X'_{i}) k\neq j \end{matrix}\right.$

since

$$\begin{matrix} \frac{\partial P_{ij}}{\partial\mu_{k}}\text{=}\frac{\partial}{\partial\mu_{k}}\left[\frac{p_{ij}e^{-\frac{1}{2}(y_{i}-\mu_{j}-B'X'_{i})'\Sigma^{-1}(y_{i}-\mu_{j}-B'X'_{i})}}{\sum_{j'=1}^{J} p_{ij'}e^{-\frac{1}{2}(y_{i}-\mu_{j'}-B'X'_{i})'\Sigma^{-1}(y_{i}-\mu_{j'}-B'X'_{i})}}\right] \\ \text{=}\frac{\sum_{j'=1}^{J} p_{ij'}e^{-\frac{1}{2}(y_{i}-\mu_{j'}-B'X'_{i})'\Sigma^{-1}(y_{i}-\mu_{j'}-B'X'_{i})}\frac{\partial p_{ij}e^{-\frac{1}{2}(y_{i}-\mu_{j}-B'X'_{i})'\Sigma^{-1}(y_{i}-\mu_{j}-B'X'_{i})}}{\partial\mu_{k}}}{\left[\sum_{j'=1}^{J} p_{ij}e^{-\frac{1}{2}(y_{i}-\mu_{j'}-B'X'_{i})'\Sigma^{-1}(y_{i}-\mu_{j'}-B'X'_{i})}\right]^{2}} \\ -\frac{p_{ij}e^{-\frac{1}{2}(y_{i}-\mu_{j}-B'X'_{i})'\Sigma^{-1}(y_{i}-\mu_{j}-B'X'_{i})}\frac{\partial}{\partial\mu_{k}}\left[\sum_{j'=1}^{J} p_{ij'}e^{-\frac{1}{2}(y_{i}-\mu_{j'}-B'X'_{i})'\Sigma^{-1}(y_{i}-\mu_{j'}-B'X'_{i})}\right]}{\left[\sum_{j'=1}^{J} p_{ij'}e^{-\frac{1}{2}(y_{i}-\mu_{j'}-B'X'_{i})'\Sigma^{-1}(y_{i}-\mu_{j'}-B'X'_{i})}\right]^{2}} \\ =P_{ij}\frac{\partial\left[-\frac{1}{2}(y_{i}-\mu_{j}-B'X'_{i})'\Sigma^{-1}(y_{i}-\mu_{j}-B'X'_{i})\right]}{\partial\mu_{k}}-P_{ij}\sum_{j'=1}^{J} P_{ij'}\frac{\partial\left[-\frac{1}{2}(y_{i}-\mu_{j'}-B'X'_{i})'\Sigma^{-1}(y_{i}-\mu_{j'}-B'X'_{i})\right]}{\partial\mu_{k}} \\ =\left\{\begin{matrix} (1-P_{ij})P_{ij}\Sigma^{-1}(y_{i}-\mu_{j}-B'X'_{i}) k=j \\ -P_{ij}P_{ik}\Sigma^{-1}(y_{i}-\mu_{k}-B'X'_{i}) k\neq j \end{matrix}\right. \end{matrix}$$

$$\begin{matrix} \frac{\partial}{\partial B}\left(\frac{\partial\ln L}{\partial\mu_{j}}\right)=\Sigma^{-1}\sum_{i=1}^{n} \frac{\partial}{\partial B}\left(P_{ij}(y_{i}-\mu_{j}-B'X'_{i})\right) \\ =\Sigma^{-1}\sum_{i=1}^{n} \left[(y_{i}-\mu_{j}-B'X'_{i})\mathrm{vec}\left(\frac{\partial P_{ij}}{\partial B}\right)^{'}+P_{ij}\frac{\partial(y_{i}-\mu_{j}-B'X'_{i})}{\partial B}\right] \\ =\Sigma^{-1}\left[\sum_{i=1}^{n} (y_{i}-\mu_{j}-B'X'_{i})\mathrm{vec}\left(\frac{\partial P_{ij}}{\partial B}\right)^{'}-I_{m}\otimes\sum_{i=1}^{n} P_{ij}X_{i}\right] \end{matrix}$$

where $\frac{\partial P_{ij}}{\partial B}\text{=}-P_{ij}X'_{i}\left(\mu_{j}-\sum_{j'=1}^{J} P_{ij'}\mu_{j'}\right)^{'}\Sigma^{-1}$ since

$$\begin{matrix} \frac{\partial P_{ij}}{\partial B}\text{=}\frac{\partial}{\partial B}\left[\frac{p_{ij}e^{-\frac{1}{2}(y_{i}-\mu_{j}-B'X'_{i})'\Sigma^{-1}(y_{i}-\mu_{j}-B'X'_{i})}}{\sum_{j'=1}^{J} p_{ij'}e^{-\frac{1}{2}(y_{i}-\mu_{j'}-B'X'_{i})'\Sigma^{-1}(y_{i}-\mu_{j'}-B'X'_{i})}}\right] \\ \text{=}\frac{\sum_{j'=1}^{J} p_{ij'}e^{-\frac{1}{2}(y_{i}-\mu_{j'}-B'X'_{i})'\Sigma^{-1}(y_{i}-\mu_{j'}-B'X'_{i})}\frac{\partial p_{ij}e^{-\frac{1}{2}(y_{i}-\mu_{j}-B'X'_{i})'\Sigma^{-1}(y_{i}-\mu_{j}-B'X'_{i})}}{\partial B}}{\left[\sum_{j'=1}^{J} p_{ij}e^{-\frac{1}{2}(y_{i}-\mu_{j'}-B'X'_{i})'\Sigma^{-1}(y_{i}-\mu_{j'}-B'X'_{i})}\right]^{2}} \\ -\frac{p_{ij}e^{-\frac{1}{2}(y_{i}-\mu_{j}-B'X'_{i})'\Sigma^{-1}(y_{i}-\mu_{j}-B'X'_{i})}\frac{\partial}{\partial B}\left[\sum_{j'=1}^{J} p_{ij'}e^{-\frac{1}{2}(y_{i}-\mu_{j'}-B'X'_{i})'\Sigma^{-1}(y_{i}-\mu_{j'}-B'X'_{i})}\right]}{\left[\sum_{j'=1}^{J} p_{ij'}e^{-\frac{1}{2}(y_{i}-\mu_{j'}-B'X'_{i})'\Sigma^{-1}(y_{i}-\mu_{j'}-B'X'_{i})}\right]^{2}} \\ =P_{ij}\frac{\partial\left[-\frac{1}{2}(y_{i}-\mu_{j}-B'X'_{i})'\Sigma^{-1}(y_{i}-\mu_{j}-B'X'_{i})\right]}{\partial B}-P_{ij}\sum_{j'=1}^{J} P_{ij'}\frac{\partial\left[-\frac{1}{2}(y_{i}-\mu_{j'}-B'X'_{i})'\Sigma^{-1}(y_{i}-\mu_{j'}-B'X'_{i})\right]}{\partial B} \\ \text{=}P_{ij}X'_{i}(y_{i}-\mu_{j}-B'X'_{i})'\Sigma^{-1}-P_{ij}\sum_{j'=1}^{J} P_{ij'}X'_{i}(y_{i}-\mu_{j'}-B'X'_{i})'\Sigma^{-1} \\ =-P_{ij}X'_{i}\left(\mu_{j}-\sum_{j'=1}^{J} P_{ij'}\mu_{j'}\right)^{'}\Sigma^{-1} \end{matrix}$$

$$\begin{matrix} \frac{\partial}{\partial\sigma_{st}}\left(\frac{\partial\ln L}{\partial\mu_{j}}\right)=\frac{\partial}{\partial\sigma_{st}}\left[\Sigma^{-1}\sum_{i=1}^{n} P_{ij}(y_{i}-\mu_{j}-B'X'_{i})\right] \\ =\frac{\partial\Sigma^{-1}}{\partial\sigma_{st}}\sum_{i=1}^{n} P_{ij}(y_{i}-\mu_{j}-B'X'_{i})+\Sigma^{-1}\sum_{i=1}^{n} \frac{\partial P_{ij}}{\partial\sigma_{st}}(y_{i}-\mu_{j}-B'X'_{i}) \end{matrix}$$

where

$$\frac{\partial\Sigma^{-1}}{\partial\sigma_{st}}=-\Sigma^{-1}\frac{\partial\Sigma}{\partial\sigma_{st}}\Sigma^{-1}=\left\{\begin{matrix} -\Sigma^{-1}E_{ss}\Sigma^{-1} s=t \\ -\Sigma^{-1}(E_{st}+E_{ts})\Sigma^{-1} s\neq t \end{matrix}\right.$$

$$\begin{matrix} \frac{\partial P_{ij}}{\partial\Sigma}\text{=}\frac{\partial}{\partial\Sigma}\left[\frac{p_{ij}e^{-\frac{1}{2}(y_{i}-\mu_{j}-B'X'_{i})'\Sigma^{-1}(y_{i}-\mu_{j}-B'X'_{i})}}{\sum_{j'=1}^{J} p_{ij'}e^{-\frac{1}{2}(y_{i}-\mu_{j'}-B'X'_{i})'\Sigma^{-1}(y_{i}-\mu_{j'}-B'X'_{i})}}\right] \\ \text{=}\frac{\sum_{j'=1}^{J} p_{ij'}e^{-\frac{1}{2}(y_{i}-\mu_{j'}-B'X'_{i})'\Sigma^{-1}(y_{i}-\mu_{j'}-B'X'_{i})}\frac{\partial p_{ij}e^{-\frac{1}{2}(y_{i}-\mu_{j}-B'X'_{i})'\Sigma^{-1}(y_{i}-\mu_{j}-B'X'_{i})}}{\partial\Sigma}}{\left[\sum_{j'=1}^{J} p_{ij}e^{-\frac{1}{2}(y_{i}-\mu_{j'}-B'X'_{i})'\Sigma^{-1}(y_{i}-\mu_{j'}-B'X'_{i})}\right]^{2}} \\ -\frac{p_{ij}e^{-\frac{1}{2}(y_{i}-\mu_{j}-B'X'_{i})'\Sigma^{-1}(y_{i}-\mu_{j}-B'X'_{i})}\frac{\partial}{\partial\Sigma}\left[\sum_{j'=1}^{J} p_{ij'}e^{-\frac{1}{2}(y_{i}-\mu_{j'}-B'X'_{i})'\Sigma^{-1}(y_{i}-\mu_{j'}-B'X'_{i})}\right]}{\left[\sum_{j'=1}^{J} p_{ij'}e^{-\frac{1}{2}(y_{i}-\mu_{j'}-B'X'_{i})'\Sigma^{-1}(y_{i}-\mu_{j'}-B'X'_{i})}\right]^{2}} \\ =P_{ij}\frac{\partial\left[-\frac{1}{2}(y_{i}-\mu_{j}-B'X'_{i})'\Sigma^{-1}(y_{i}-\mu_{j}-B'X'_{i})\right]}{\partial\Sigma}-P_{ij}\sum_{j'=1}^{J} P_{ij'}\frac{\partial\left[-\frac{1}{2}(y_{i}-\mu_{j'}-B'X'_{i})'\Sigma^{-1}(y_{i}-\mu_{j'}-B'X'_{i})\right]}{\partial\Sigma} \\ =P_{ij}\left\{\Sigma^{-1}(y_{i}-\mu_{j}-B'X'_{i})(y_{i}-\mu_{j}-B'X'_{i})'\Sigma^{-1}-\frac{1}{2}\mathrm{diag}\left[\Sigma^{-1}(y_{i}-\mu_{j}-B'X'_{i})(y_{i}-\mu_{j}-B'X'_{i})'\Sigma^{-1}\right]\right\} \\ -P_{ij}\left\{\sum_{j'=1}^{J} P_{ij'}\left[\Sigma^{-1}(y_{i}-\mu_{j'}-B'X'_{i})(y_{i}-\mu_{j}^{'}-B'X'_{i})'\Sigma^{-1}-\frac{1}{2}\mathrm{diag}\left[\Sigma^{-1}(y_{i}-\mu_{j'}-B'X'_{i})(y_{i}-\mu_{j}^{'}-B'X'_{i})'\Sigma^{-1}\right]\right]\right\} \end{matrix}$$

$$\begin{matrix} \frac{\partial}{\partial B}\left(\frac{\partial\ln L}{\partial B}\right)\text{=}-\sum_{j=1}^{J} \frac{\partial}{\partial B}X'P_{j}\mu'_{j}\Sigma^{-1}-\frac{\partial X'XB\Sigma^{-1}}{\partial B} \\ = -\sum_{j=1}^{J} \Sigma^{-1}\mu_{j}\otimes X'\left(\mathrm{vec}\left(\frac{\partial P_{1j}}{\partial B}\right),\mathrm{vec}\left(\frac{\partial P_{2j}}{\partial B}\right),\cdots,\mathrm{vec}\left(\frac{\partial P_{nj}}{\partial B}\right)\right)^{'}-\Sigma^{-1}\otimes X'X \end{matrix}$$

where$\frac{\partial P_{ij}}{\partial B}\text{=}-P_{ij}X'_{i}\left(\mu_{j}-\sum_{j'=1}^{J} P_{ij'}\mu_{j'}\right)^{'}\Sigma^{-1}$

$$\begin{matrix} \frac{\partial}{\partial\sigma_{st}}\left(\frac{\partial\ln L}{\partial B}\right)=\frac{\partial}{\partial\sigma_{st}}\left[X'\left(Y-\sum_{j=1}^{J} P_{j}\otimes\mu'_{j}\right)-X'XB\right]\Sigma^{-1} \\ \text{=}-X'\sum_{j=1}^{J} \frac{\partial P_{j}}{\partial\sigma_{st}}\mu'_{j}\Sigma^{-1}+X'\left(Y-\sum_{j=1}^{J} P_{j}\mu'_{j}-XB\right)\frac{\partial\Sigma^{-1}}{\partial\sigma_{st}} \end{matrix}$$

Where

$$\frac{\partial\Sigma^{-1}}{\partial\sigma_{st}}=-\Sigma^{-1}\frac{\partial\Sigma}{\partial\sigma_{st}}\Sigma^{-1}=\left\{\begin{matrix} -\Sigma^{-1}E_{ss}\Sigma^{-1} s=t \\ -\Sigma^{-1}(E_{st}+E_{ts})\Sigma^{-1} s\neq t \end{matrix}\right.$$

$$\begin{matrix} \frac{\partial P_{ij}}{\partial\Sigma}\text{=}=P_{ij}\left\{\Sigma^{-1}(y_{i}-\mu_{j}-B'X'_{i})(y_{i}-\mu_{j}-B'X'_{i})'\Sigma^{-1}-\frac{1}{2}\mathrm{diag}\left[\Sigma^{-1}(y_{i}-\mu_{j}-B'X'_{i})(y_{i}-\mu_{j}-B'X'_{i})'\Sigma^{-1}\right]\right\} \\ -P_{ij}\left\{\sum_{j'=1}^{J} P_{ij'}\left[\Sigma^{-1}(y_{i}-\mu_{j'}-B'X'_{i})(y_{i}-\mu_{j}^{'}-B'X'_{i})'\Sigma^{-1}-\frac{1}{2}\mathrm{diag}\left[\Sigma^{-1}(y_{i}-\mu_{j'}-B'X'_{i})(y_{i}-\mu_{j}^{'}-B'X'_{i})'\Sigma^{-1}\right]\right]\right\} \end{matrix}$$

$$\begin{matrix} \frac{\partial}{\partial\sigma_{st}}\left(\frac{\partial\ln L}{\partial\Sigma}\right)=-n\frac{\partial\Sigma^{-1}}{\partial\sigma_{st}}+\frac{n}{2}\mathrm{diag}\left(\frac{\partial\Sigma^{-1}}{\partial\sigma_{st}}\right) \\ +\sum_{i=1}^{n} \sum_{j=1}^{J} \left\{\frac{\partial P_{ij}}{\partial\sigma_{st}}\left[\Sigma^{-1}(y_{i}-\mu_{j}-B'X'_{i})(y_{i}-\mu_{j}-B'X'_{i})'\Sigma^{-1}\right.\right. \\ \left.-\frac{1}{2}\mathrm{diag}\left(\Sigma^{-1}(y_{i}-\mu_{j}-B'X'_{i})(y_{i}-\mu_{j}-B'X'_{i})'\Sigma^{-1}\right)\right] \\ +P_{ij}\left[\frac{\partial\Sigma^{-1}}{\partial\sigma_{st}}(y_{i}-\mu_{j}-B'X'_{i})(y_{i}-\mu_{j}-B'X'_{i})'\Sigma^{-1}\right. \\ \left.-\frac{1}{2}\mathrm{diag}\left(\frac{\partial\Sigma^{-1}}{\partial\sigma_{st}}(y_{i}-\mu_{j}-B'X'_{i})(y_{i}-\mu_{j}-B'X'_{i})'\Sigma^{-1}\right)\right] \\ +P_{ij}\left[\Sigma^{-1}(y_{i}-\mu_{j}-B'X'_{i})(y_{i}-\mu_{j}-B'X'_{i})'\frac{\partial\Sigma^{-1}}{\partial\sigma_{st}}\right. \\ \left.\left.-\frac{1}{2}\mathrm{diag}\left(\Sigma^{-1}(y_{i}-\mu_{j}-B'X'_{i})(y_{i}-\mu_{j}-B'X'_{i})'\frac{\partial\Sigma^{-1}}{\partial\sigma_{st}}\right)\right]\right\} \end{matrix}$$

where

$$\frac{\partial\Sigma^{-1}}{\partial\sigma_{st}}=-\Sigma^{-1}\frac{\partial\Sigma}{\partial\sigma_{st}}\Sigma^{-1}=\left\{\begin{matrix} -\Sigma^{-1}E_{ss}\Sigma^{-1} s=t \\ -\Sigma^{-1}(E_{st}+E_{ts})\Sigma^{-1} s\neq t \end{matrix}\right.$$

$$\begin{matrix} \frac{\partial P_{ij}}{\partial\Sigma}\text{=}P_{ij}\sum_{j'=1}^{J} P_{ij'}\Sigma^{-1}\left[(y_{i}-B'X'_{i})(\mu_{j'}-\mu_{j})'+(\mu_{j'}-\mu_{j})(y_{i}-B'X'_{i})'+\mu_{j}\mu'_{j}-\mu_{j'}\mu'_{j'}\right]\Sigma^{-1} \\ -\frac{1}{2}P_{ij}\sum_{j'=1}^{J} P_{ij'}\mathrm{diag}\left\{\Sigma^{-1}\left[(y_{i}-B'X'_{i})(\mu_{j'}-\mu_{j})'+(\mu_{j'}-\mu_{j})(y_{i}-B'X'_{i})'+\mu_{j}\mu'_{j}-\mu_{j'}\mu'_{j'}\right]\Sigma^{-1}\right\} \end{matrix}$$

$$\begin{matrix} \frac{\partial P_{ij}}{\partial\Sigma}\text{=}=P_{ij}\left\{\Sigma^{-1}(y_{i}-\mu_{j}-B'X'_{i})(y_{i}-\mu_{j}-B'X'_{i})'\Sigma^{-1}-\frac{1}{2}\mathrm{diag}\left[\Sigma^{-1}(y_{i}-\mu_{j}-B'X'_{i})(y_{i}-\mu_{j}-B'X'_{i})'\Sigma^{-1}\right]\right\} \\ -P_{ij}\left\{\sum_{j'=1}^{J} P_{ij'}\left[\Sigma^{-1}(y_{i}-\mu_{j'}-B'X'_{i})(y_{i}-\mu_{j}^{'}-B'X'_{i})'\Sigma^{-1}-\frac{1}{2}\mathrm{diag}\left[\Sigma^{-1}(y_{i}-\mu_{j'}-B'X'_{i})(y_{i}-\mu_{j}^{'}-B'X'_{i})'\Sigma^{-1}\right]\right]\right\} \end{matrix}$$
