# Supplementary material for: MVQTLCIM: composite interval mapping of multivariate traits in a hybrid F1 population of outbred species
Source: BMC Bioinformatics. 2017 Nov 23;18:515. doi: 10.1186/s12859-017-1908-1 (PMC5701343; doi:10.1186/s12859-017-1908-1)
Supplement: Supplementary file 2 — Average of parameter estimates with the standard deviation in bracket under different sample sizes when the QTL segregation type is QQ × Qq, based on 1000 simulation replicates. Table S2. Average of parameter estimates with the standard deviation in bracket under different sample sizes when the QTL segregation type is Qq × Qq, based on 1000 simulation replicates. Table S3. Average of parameter estimates with the standard deviation in bracket under different sample sizes when the QTL segregation type is Q1Q2 × Q3Q4, based on 1000 simulation replicates. Table S4. Average of parameter estimates with the standard deviation in bracket under different sample sizes when the QTL segregation type is Qq × QQ, based on 1000 simulation replicates. Table S5. Average of parameter estimates with the standard deviation in bracket under different sample sizes when the QTL segregation type is Qq × qQ, based on 1000 simulation replicates. Table S6. Summary on average estimates of QTL heritabilities (%) with the standard deviation in brackets under different time points (T1-T8) and different sample sizes based on 1000 simulation replicates. Table S7. The average estimate of the residual covariance matrix with standard deviations in brackets when the sample size is 300 and the QTL segregation type is Qq × qQ, based on 1000 simulation replicates. (DOCX 42 kb) [file 12859_2017_1908_MOESM2_ESM.docx]

**Table S1.** Average of parameter estimates with the standard deviation in bracket under different sample sizes when the QTL segregation type is *QQ*×*Qq*, based on 1000 simulation replicates.

| Value  Type | Sample Size | QTL  Genotype | T1 | T2 | T3 | T4 | T5 | T6 | T7 | T8 | QTL  Position |
| --- | --- | --- | --- | --- | --- | --- | --- | --- | --- | --- | --- |
| True |  | *QQ* | -1.21 | 1.95 | 3.94 | 7.44 | 9.89 | 11.43 | 12.34 | 12.86 | 5.0 |
|  |  | *Qq* | 1.21 | -1.95 | -3.94 | -7.44 | -9.89 | -11.43 | -12.34 | -12.86 |  |
|  |  |  |  |  |  |  |  |  |  |  |  |
| Estimate | 300 | *QQ* | -1.16  (0.51) | 2.04  (0.73) | 4.05  (0.79) | 7.58  (0.87) | 10.06  (0.93) | 11.61  (0.97) | 12.52  (0.99) | 13.04  (1.01) | 4.99  (1.26) |
|  |  | *Qq* | 1.16  (0.51) | -2.04  (0.73) | -4.05  (0.79) | -7.58  (0.87) | -10.06  (0.93) | -11.61  (0.97) | -12.52  (0.99) | -13.04  (1.01) |  |
|  |  |  |  |  |  |  |  |  |  |  |  |
|  | 200 | *QQ* | -1.18  (0.62) | 2.02  (0.90) | 4.02  (0.97) | 7.54  (1.08) | 10.04  (1.17) | 11.61  (1.21) | 12.53  (1.24) | 13.05  (1.25) | 5.11  (1.51) |
|  |  | *Qq* | 1.18  (0.62) | -2.02  (0.90) | -4.02  (0.97) | -7.54  (1.08) | -10.04  (1.17) | -11.61  (1.21) | -12.53  (1.24) | -13.05  (1.25) |  |
|  |  |  |  |  |  |  |  |  |  |  |  |
|  | 150 | *QQ* | -1.21  (0.74) | 1.96  (1.07) | 3.96  (1.19) | 7.48  (1.34) | 9.96  (1.42) | 11.51  (1.49) | 12.41  (1.51) | 12.93  (1.53) | 4.97  (1.76) |
|  |  | *Qq* | 1.21  (0.74) | -1.96  (1.07) | -3.96  (1.19) | -7.48  (1.34) | -9.96  (1.42) | -11.51  (1.49) | -12.41  (1.51) | -12.93  (1.53) |  |

**Table S2.** Average of parameter estimates with the standard deviation in bracket under different sample sizes when the QTL segregation type is *Qq*×*Qq*, based on 1000 simulation replicates.

| Value  Type | Sample Size | QTL  Genotype | T1 | T2 | T3 | T4 | T5 | T6 | T7 | T8 | Position |
| --- | --- | --- | --- | --- | --- | --- | --- | --- | --- | --- | --- |
| True |  | *QQ* | 3.47 | 8.69 | 11.13 | 15.02 | 17.57 | 19.13 | 20.05 | 20.57 | 5.0 |
|  |  | *Qq* | -0.81 | -1.19 | -1.35 | -1.65 | -1.89 | -2.07 | -2.19 | -2.26 |  |
|  |  | *qq* | -1.84 | -6.30 | -8.43 | -11.72 | -13.79 | -15.00 | -15.67 | -16.04 |  |
|  |  |  |  |  |  |  |  |  |  |  |  |
| Estimate | 300 | *QQ* | 3.50  (0.93) | 8.83  (1.35) | 11.34  (1.47) | 15.35  (1.64) | 17.99  (1.76) | 19.62  (1.84) | 20.57  (1.90) | 21.12  (1.94) | 5.33  (1.62) |
|  |  | *Qq* | -0.81  (0.54) | -1.20  (0.78) | -1.37  (0.86) | -1.68  (0.98) | -1.95  (1.05) | -2.14  (1.11) | -2.26  (1.14) | -2.34  (1.15) |  |
|  |  | *qq* | -1.88  (0.87) | -6.43  (1.27) | -8.61  (1.40) | -11.98  (1.60) | -14.09  (1.74) | -15.34  (1.83) | -16.05  (1.87) | -16.44  (1.94) |  |
|  |  |  |  |  |  |  |  |  |  |  |  |
|  | 200 | *QQ* | 3.49  (1.19) | 8.83  (1.72) | 11.33  (1.85) | 15.31  (2.07) | 17.96  (2.19) | 19.54  (2.32) | 20.50  (2.35) | 21.08  (2.42) | 5.31  (2.09) |
|  |  | *Qq* | -0.79  (0.71) | -1.19  (1.00) | -1.35  (1.09) | -1.67  (1.22) | -1.93  (1.29) | -2.11  (1.34) | -2.25  (1.37) | -2.31  (1.39) |  |
|  |  | *qq* | -1.90  (1.18) | -6.45  (1.69) | -8.63  (1.84) | -11.98  (2.07) | -14.10  (2.23) | -15.32  (2.32) | -16.00  (2.40) | -16.45  (2.44) |  |
|  |  |  |  |  |  |  |  |  |  |  |  |
|  | 150 | *QQ* | 3.56  (1.39) | 8.85  (2.00) | 11.34  (2.17) | 15.29  (2.44) | 17.90  (2.65) | 19.47  (2.79) | 20.40  (2.89) | 20.94  (2.95) | 5.19  (2.51) |
|  |  | *Qq* | -0.87  (0.86) | -1.26  (1.21) | -1.42  (1.30) | -1.74  (1.44) | -2.00  (1.56) | -2.18  (1.61) | -2.30  (1.66) | -2.38  (1.71) |  |
|  |  | *qq* | -1.82  (1.40) | -6.33  (2.00) | -8.49  (2.16) | -11.80  (2.40) | -13.91  (2.61) | -15.12  (2.73) | -15.80  (2.83) | -16.17  (2.86) |  |

**Table S3.** Average of parameter estimates with the standard deviation in bracket under different sample sizes when the QTL segregation type is *Q*­_1_*Q*_2_×*Q*­_3_*Q*_4_, based on 1000 simulation replicates.

| Value  Type | Sample Size | QTL  Genotype | T1 | T2 | T3 | T4 | T5 | T6 | T7 | T8 | Position |
| --- | --- | --- | --- | --- | --- | --- | --- | --- | --- | --- | --- |
| True |  | *Q*_1_*Q*_3_ | 5.26 | 11.16 | 14.41 | 20.44 | 25.13 | 28.36 | 30.45 | 31.73 | 3.0 |
|  |  | *Q*_1_*Q*_4_ | 2.27 | 3.32 | 3.07 | 1.86 | 0.52 | -0.53 | -1.25 | -1.71 |  |
|  |  | *Q*_2_*Q*_3_ | -8.94 | -12.78 | -13.87 | -15.38 | -16.44 | -17.18 | -17.68 | -18.01 |  |
|  |  | *Q*_2_*Q*_4_ | 1.40 | -1.70 | -3.61 | -6.92 | -9.21 | -10.65 | -11.52 | -12.02 |  |
|  |  |  |  |  |  |  |  |  |  |  |  |
| Estimate | 300 | *Q*_1_*Q*_3_ | 5.29  (0.75) | 11.31  (1.09) | 14.59  (1.19) | 20.70  (1.34) | 25.46  (1.48) | 28.72  (1.58) | 30.81  (1.64) | 32.13  (1.66) | 3.32  (1.31) |
|  |  | *Q*_1_*Q*_4_ | 2.17  (0.81) | 3.10  (1.19) | 2.82  (1.33) | 1.56  (1.53) | 0.19  (1.67) | -0.89  (1.76) | -1.59  (1.81) | -2.06  (1.86) |  |
|  |  | *Q*_2_*Q*_3_ | -8.90  (0.82) | -12.72  (1.19) | -13.78  (1.32) | -15.27  (1.53) | -16.32  (1.66) | -17.04  (1.77) | -17.55  (1.83) | -17.88  (1.86) |  |
|  |  | *Q*_2_*Q*_4_ | 1.44  (0.79) | -1.70  (1.14) | -3.63  (1.27) | -7.00  (1.44) | -9.33  (1.59) | -10.79  (1.68) | -11.67  (1.78) | -12.19  (1.79) |  |
|  |  |  |  |  |  |  |  |  |  |  |  |
|  | 200 | *Q*_1_*Q*_3_ | 5.29  (0.94) | 11.31  (1.35) | 14.63  (1.51) | 20.73  (1.73) | 25.45  (1.91) | 28.69  (2.03) | 30.77  (2.13) | 32.04  (2.17) | 3.33  (1.73) |
|  |  | *Q*_1_*Q*_4_ | 2.22  (1.09) | 3.17  (1.56) | 2.86  (1.70) | 1.57  (1.94) | 0.21  (2.10) | -0.85  (2.21) | -1.56  (2.30) | -2.06  (2.36) |  |
|  |  | *Q*_2_*Q*_3_ | -8.90  (1.10) | -12.73  (1.60) | -13.79  (1.75) | -15.26  (1.98) | -16.30  (2.14) | -17.05  (2.25) | -17.55  (2.37) | -17.81  (2.41) |  |
|  |  | *Q*_2_*Q*_4_ | 1.39  (0.97) | -1.75  (1.40) | -3.70  (1.54) | -7.05  (1.75) | -9.36  (1.91) | -10.80  (1.98) | -11.65  (2.04) | -12.17  (2.11) |  |
|  |  |  |  |  |  |  |  |  |  |  |  |
|  | 150 | *Q*_1_*Q*_3_ | 5.27  (1.18) | 11.25  (1.67) | 14.54  (1.81) | 20.62  (2.03) | 25.34  (2.21) | 28.61  (2.36) | 30.68  (2.41) | 31.97  (2.50) | 3.41  (2.11) |
|  |  | *Q*_1_*Q*_4_ | 2.22  (1.35) | 3.14  (1.96) | 2.84  (2.14) | 1.56  (2.41) | 0.19  (2.58) | -0.90  (2.71) | -1.65  (2.74) | -2.10  (2.87) |  |
|  |  | *Q*_2_*Q*_3_ | -8.84  (1.32) | -12.63  (1.93) | -13.71  (2.13) | -15.17  (2.43) | -16.18  (2.60) | -16.92  (2.78) | -17.36  (2.89) | -17.66  (2.94) |  |
|  |  | *Q*_2_*Q*_4_ | 1.36  (1.23) | -1.76  (1.80) | -3.67  (1.97) | -7.01  (2.26) | -9.35  (2.47) | -10.78  (2.59) | -11.67  (2.73) | -12.21  (2.79) |  |

**Table S4.** Average of parameter estimates with the standard deviation in bracket under different sample sizes when the QTL segregation type is *Qq*×*QQ*, based on 1000 simulation replicates.

| Value  Type | Sample Size | QTL  Genotype | T1 | T2 | T3 | T4 | T5 | T6 | T7 | T8 | Position |
| --- | --- | --- | --- | --- | --- | --- | --- | --- | --- | --- | --- |
| True |  | *QQ* | -7.38 | -9.66 | -9.78 | -9.31 | -8.69 | -8.23 | -7.93 | -7.76 | 8.0 |
|  |  | *Qq* | 7.38 | 9.66 | 9.78 | 9.31 | 8.69 | 8.23 | 7.93 | 7.76 |  |
|  |  |  |  |  |  |  |  |  |  |  |  |
| Estimate | 300 | *QQ* | -7.31  (0.41) | -9.56  (0.64) | -9.68  (0.73) | -9.21  (0.88) | -8.58  (0.98) | -8.14  (1.05) | -7.84  (1.10) | -7.66  (1.12) | 7.17  (2.20) |
|  |  | *Qq* | 7.31  (0.41) | 9.56  (0.64) | 9.68  (0.73) | 9.21  (0.88) | 8.58  (0.98) | 8.14  (1.05) | 7.84  (1.10) | 7.66  (1.12) |  |
|  |  |  |  |  |  |  |  |  |  |  |  |
|  | 200 | *QQ* | -7.31  (0.56) | -9.54  (0.88) | -9.66  (1.01) | -9.21  (1.19) | -8.58  (1.33) | -8.09  (1.42) | -7.79  (1.47) | -7.61  (1.50) | 6.67  (2.85) |
|  |  | *Qq* | 7.31  (0.56) | 9.54  (0.88) | 9.66  (1.01) | 9.21  (1.19) | 8.58  (1.33) | 8.09  (1.42) | 7.79  (1.47) | 7.61  (1.50) |  |
|  |  |  |  |  |  |  |  |  |  |  |  |
|  | 150 | *QQ* | -7.30  (0.64) | -9.50  (0.99) | -9.62  (1.15) | -9.13  (1.38) | -8.51  (1.55) | -8.05  (1.64) | -7.76  (1.71) | -7.60  (1.73) | 6.69  (3.06) |
|  |  | *Qq* | 7.30  (0.64) | 9.50  (0.99) | 9.62  (1.15) | 9.13  (1.38) | 8.51  (1.55) | 8.05  (1.64) | 7.76  (1.71) | 7.60  (1.73) |  |

**Table S5.** Average of parameter estimates with the standard deviation in bracket under different sample sizes when the QTL segregation type is *Qq*×*qQ*, based on 1000 simulation replicates.

| Value  Type | Sample Size | QTL  Genotype | T1 | T2 | T3 | T4 | T5 | T6 | T7 | T8 | Position |
| --- | --- | --- | --- | --- | --- | --- | --- | --- | --- | --- | --- |
| True |  | *QQ* | -6.42 | -10.12 | -11.27 | -12.65 | -13.34 | -13.66 | -13.82 | -13.89 | 7.0 |
|  |  | *Qq* | -0.99 | -2.52 | -3.21 | -4.26 | -4.90 | -5.25 | -5.44 | -5.54 |  |
|  |  | *qq* | 8.40 | 15.16 | 17.70 | 21.18 | 23.13 | 24.17 | 24.71 | 24.98 |  |
|  |  |  |  |  |  |  |  |  |  |  |  |
| Estimate | 300 | *QQ* | -6.44  (0.92) | -10.13  (1.32) | -11.28  (1.44) | -12.67  (1.61) | -13.36  (1.73) | -13.69  (1.83) | -13.85  (1.92) | -13.93  (1.96) | 6.55  (1.70) |
|  |  | *Qq* | -0.98  (0.52) | -2.55  (0.75) | -3.26  (0.82) | -4.37  (0.92) | -5.04  (1.00) | -5.41  (1.05) | -5.62  (1.09) | -5.73  (1.11) |  |
|  |  | *qq* | 8.39  (0.91) | 15.23  (1.31) | 17.81  (1.43) | 21.41  (1.62) | 23.43  (1.77) | 24.50  (1.84) | 25.09  (1.89) | 25.38  (1.92) |  |
|  |  |  |  |  |  |  |  |  |  |  |  |
|  | 200 | *QQ* | -6.48  (1.17) | -10.21  (1.68) | -11.40  (1.84) | -12.79  (2.08) | -13.47  (2.25) | -13.79  (2.35) | -13.92  (2.39) | -14.00  (2.43) | 6.55  (2.03) |
|  |  | *Qq* | -0.98  (0.68) | -2.55  (0.97) | -3.26  (1.05) | -4.35  (1.20) | -5.02  (1.28) | -5.41  (1.33) | -5.61  (1.38) | -5.71  (1.41) |  |
|  |  | *qq* | 8.45  (1.12) | 15.30  (1.63) | 17.91  (1.78) | 21.49  (2.03) | 23.50  (2.17) | 24.61  (2.30) | 25.14  (2.40) | 25.42  (2.48) |  |
|  |  |  |  |  |  |  |  |  |  |  |  |
|  | 150 | *QQ* | -6.45  (1.42) | -10.16  (2.06) | -11.30  (2.24) | -12.68  (2.51) | -13.36  (2.71) | -13.69  (2.86) | -13.86  (2.98) | -13.94  (3.03) | 6.15  (2.52) |
|  |  | *Qq* | -0.95  (0.80) | -2.49  (1.16) | -3.21  (1.26) | -4.31  (1.41) | -4.97  (1.54) | -5.35  (1.62) | -5.57  (1.71) | -5.67  (1.74) |  |
|  |  | *qq* | 8.35  (1.35) | 15.14  (1.91) | 17.72  (2.09) | 21.30  (2.36) | 23.31  (2.56) | 24.40  (2.66) | 24.99  (2.78) | 25.28  (2.82) |  |

**Table S6.** Summary on average estimates of QTL heritabilities (%) with the standard deviation in brackets under different time points (T1-T8) and different sample sizes based on 1000 simulation replicates.

| Value  Type | Sample Size | QTL  Segregation  Pattern | T1 | T2 | T3 | T4 | T5 | T6 | T7 | T8 | Average |
| --- | --- | --- | --- | --- | --- | --- | --- | --- | --- | --- | --- |
| True |  | *QQ*×*Qq* | 1.13 | 1.19 | 3.66 | 8.71 | 11.95 | 13.70 | 14.60 | 15.07 | 8.75 |
|  |  | *Qq*×*Qq* | 3.21 | 9.21 | 11.71 | 14.49 | 15.45 | 15.72 | 15.75 | 15.74 | 12.66 |
|  |  | *Q*_1_*Q*_2_×*Q*_3_*Q*_4_ | 22.01 | 23.56 | 24.91 | 27.77 | 30.13 | 31.81 | 32.95 | 33.69 | 28.35 |
|  |  | *Qq*×*QQ* | 41.81 | 29.11 | 22.56 | 13.65 | 9.23 | 7.10 | 6.04 | 5.48 | 16.87 |
|  |  | *Qq*×*qQ* | 21.84 | 26.93 | 27.17 | 25.39 | 23.23 | 21.66 | 20.65 | 20.02 | 23.36 |
|  |  |  |  |  |  |  |  |  |  |  |  |
| Estimate | 300 | *QQ*×*Qq* | 1.24  (0.95) | 1.47  (0.97) | 4.03  (1.52) | 9.18  (2.07) | 12.49  (2.26) | 14.26  (2.36) | 15.14  (2.38) | 15.59  (2.40) | 9.17  (1.58) |
|  |  | *Qq*×*Qq* | 3.72  (1.72) | 9.92  (2.57) | 12.53  (2.69) | 15.44  (2.75) | 16.45  (2.73) | 16.77  (2.68) | 16.82  (2.64) | 16.81  (2.66) | 13.56  (2.38) |
|  |  | *Q*_1_*Q*_2_×*Q*_3_*Q*_4_ | 22.45  (3.61) | 24.07  (3.40) | 25.46  (3.31) | 28.46  (3.22) | 30.96  (3.25) | 32.68  (3.31) | 33.83  (3.31) | 34.61  (3.31) | 29.06  (3.00) |
|  |  | *Qq*×*QQ* | 41.36  (4.30) | 28.75  (3.66) | 22.30  (3.26) | 13.51  (2.53) | 9.13  (2.04) | 7.07  (1.77) | 6.03  (1.65) | 5.46  (1.56) | 16.70  (2.37) |
|  |  | *Qq*×*qQ* | 22.27  (3.81) | 27.50  (3.88) | 27.81  (3.77) | 26.14  (3.47) | 24.02  (3.24) | 22.44  (3.05) | 21.46  (2.95) | 20.82  (2.91) | 24.06  (3.13) |
|  |  |  |  |  |  |  |  |  |  |  |  |
|  | 200 | *QQ*×*Qq* | 1.36  (1.21) | 1.51  (1.16) | 4.01  (1.82) | 9.11  (2.46) | 12.47  (2.77) | 14.30  (2.86) | 15.24  (2.88) | 15.70  (2.91) | 9.21  (1.91) |
|  |  | *Qq*×*Qq* | 3.98  (2.19) | 10.14  (3.27) | 12.68  (3.45) | 15.53  (3.58) | 16.56  (3.54) | 16.81  (3.53) | 16.88  (3.47) | 16.94  (3.45) | 13.69  (3.10) |
|  |  | *Q*_1_*Q*_2_×*Q*_3_*Q*_4_ | 22.63  (4.53) | 24.30  (4.24) | 25.69  (4.16) | 28.67  (4.12) | 31.10  (4.19) | 32.83  (4.30) | 33.99  (4.34) | 34.68  (4.38) | 29.23  (3.82) |
|  |  | *Qq*×*QQ* | 41.22  (5.56) | 28.63  (4.86) | 22.23  (4.39) | 13.58  (3.40) | 9.22  (2.77) | 7.09  (2.41) | 6.05  (2.21) | 5.51  (2.07) | 16.69  (3.20) |
|  |  | *Qq*×*qQ* | 22.72  (4.69) | 27.91  (4.86) | 28.24  (4.76) | 26.51  (4.38) | 24.33  (4.08) | 22.78  (3.92) | 21.72  (3.79) | 21.08  (3.79) | 24.41  (4.01) |
|  |  |  |  |  |  |  |  |  |  |  |  |
|  | 150 | *QQ*×*Qq* | 1.54  (1.44) | 1.57  (1.43) | 4.08  (2.26) | 9.19  (3.12) | 12.51  (3.42) | 14.31  (3.56) | 15.21  (3.64) | 15.67  (3.68) | 9.26  (2.40) |
|  |  | *Qq*×*Qq* | 4.41  (2.70) | 10.42  (3.84) | 12.95  (4.06) | 15.75  (4.18) | 16.74  (4.17) | 16.95  (4.16) | 16.98  (4.14) | 16.97  (4.11) | 13.90  (3.66) |
|  |  | *Q*_1_*Q*_2_×*Q*_3_*Q*_4_ | 22.95  (5.59) | 24.66  (5.12) | 26.09  (5.00) | 29.06  (4.95) | 31.47  (4.96) | 33.22  (5.07) | 34.35  (5.12) | 35.09  (5.15) | 29.61  (4.56) |
|  |  | *Qq*×*QQ* | 41.45  (6.45) | 28.80  (5.54) | 22.39  (5.02) | 13.59  (3.92) | 9.27  (3.22) | 7.17  (2.77) | 6.13  (2.55) | 5.60  (2.40) | 16.80  (3.65) |
|  |  | *Qq*×*qQ* | 22.71  (5.88) | 28.00  (5.94) | 28.30  (5.84) | 26.62  (5.38) | 24.45  (5.02) | 22.87  (4.75) | 21.91  (4.62) | 21.29  (4.55) | 24.52  (4.87) |

**Table S7.** The average estimate of the residual covariance matrix with standard deviations in brackets when the sample size is 300 and the QTL segregation type is *Qq*×*qQ*, based on 1000 simulation replicates.

| 62.7  (5.76) | 88.23  (8.16) | 92.57  (8.73) | 96.45  (9.37) | 96.4  (9.73) | 95.1  (10.04) | 93.46  (10.28) | 91.67  (10.29) |
| --- | --- | --- | --- | --- | --- | --- | --- |
| 88.23  (8.16) | 132.55  (12.11) | 140.38  (13.1) | 149.11  (14.43) | 151.03  (15.2) | 150.18  (15.8) | 148.19  (16.21) | 145.62  (16.25) |
| 92.57  (8.73) | 140.38  (13.1) | 157.16  (14.56) | 168.63  (16.26) | 171.95  (17.22) | 171.57  (17.92) | 169.42  (18.38) | 166.46  (18.42) |
| 96.45  (9.37) | 149.11  (14.43) | 168.63  (16.26) | 195.18  (18.97) | 201.09  (20.38) | 201.66  (21.36) | 199.48  (21.95) | 195.93  (22.07) |
| 96.4  (9.73) | 151.03  (15.2) | 171.95  (17.22) | 201.09  (20.38) | 223.54  (22.73) | 224.77  (23.87) | 222.39  (24.55) | 218.09  (24.73) |
| 95.1  (10.04) | 150.18  (15.8) | 171.57  (17.92) | 201.66  (21.36) | 224.77  (23.87) | 244.08  (25.61) | 241.27  (26.37) | 236.24  (26.58) |
| 93.46  (10.28) | 148.19  (16.21) | 169.42  (18.38) | 199.48  (21.95) | 222.39  (24.55) | 241.27  (26.37) | 257.97  (27.83) | 252.05  (27.99) |
| 91.67  (10.29) | 145.62  (16.25) | 166.46  (18.42) | 195.93  (22.07) | 218.09  (24.73) | 236.24  (26.58) | 252.05  (27.99) | 266.82  (28.89) |
