# Supplementary material for: MVQTLCIM: composite interval mapping of multivariate traits in a hybrid F1 population of outbred species
Source: BMC Bioinformatics. 2017 Nov 23;18:515. doi: 10.1186/s12859-017-1908-1 (PMC5701343; doi:10.1186/s12859-017-1908-1)
Supplement: Supplementary file 4 — Biological process GO category for the genes within the region of QTL Q1D1. Figure S2. Biological process GO category for the genes within the region of QTL Q2D1. Figure S3. Biological process GO category for the genes within the region of QTL Q3D1. Figure S4. Biological process GO category for the genes within the region of QTL Q1D2. Figure S5. Biological process GO category for the genes within the region of QTL Q2D2. Figure S6. Biological process GO category for the genes within the region of QTL Q3D2. Figure S7. Biological process GO category for the genes within the region of QTL QD5. Figure S8. Biological process GO category for the genes within the region of QTL QD9. Figure S9. Biological process GO category for the genes within the region of QTL Q1D14. Figure S10. Biological process GO category for the genes within the region of QTL Q2D14. Figure S11. Biological process GO category for the genes within the region of QTL QS7. Figure S12. Biological process GO category for the genes within the region of QTL QS9. (DOCX 642 kb) [file 12859_2017_1908_MOESM4_ESM.docx]

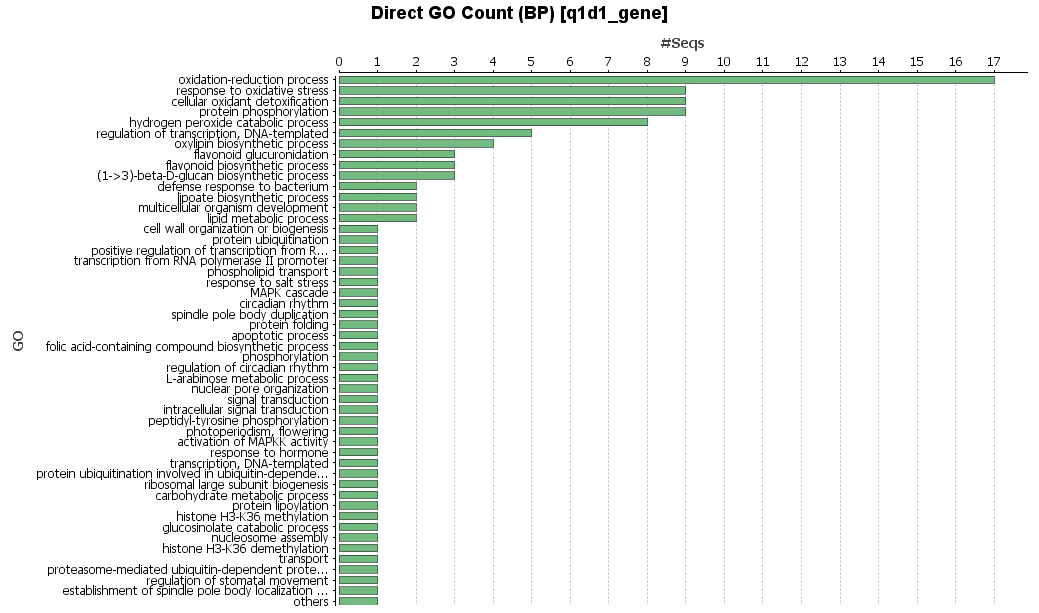


**Fig. S1** Biological process GO category for the genes within the region of QTL Q1D1.


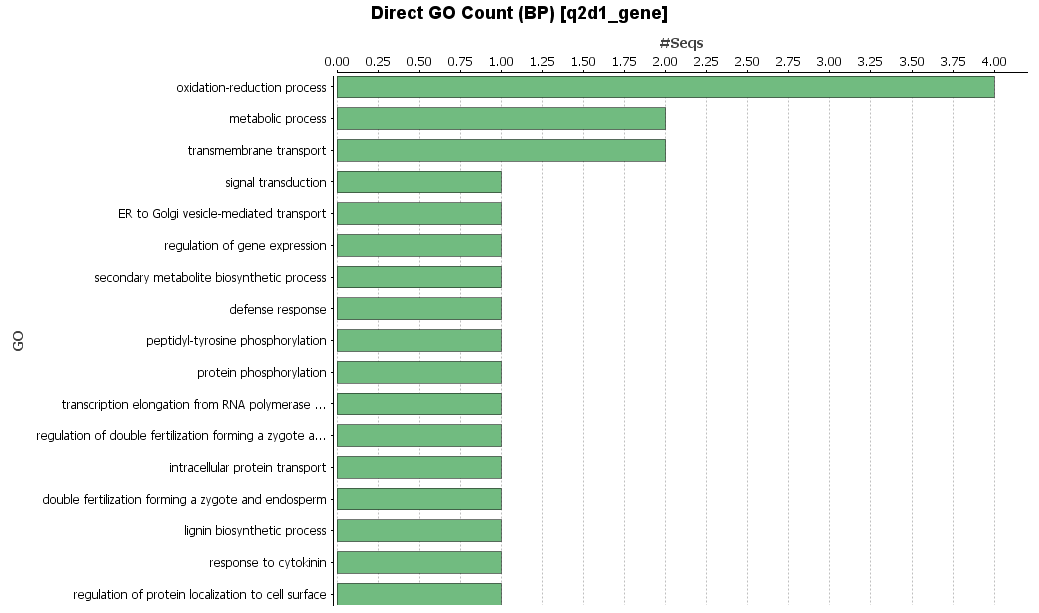


**Fig. S2** Biological process GO category for the genes within the region of QTL Q2D1.


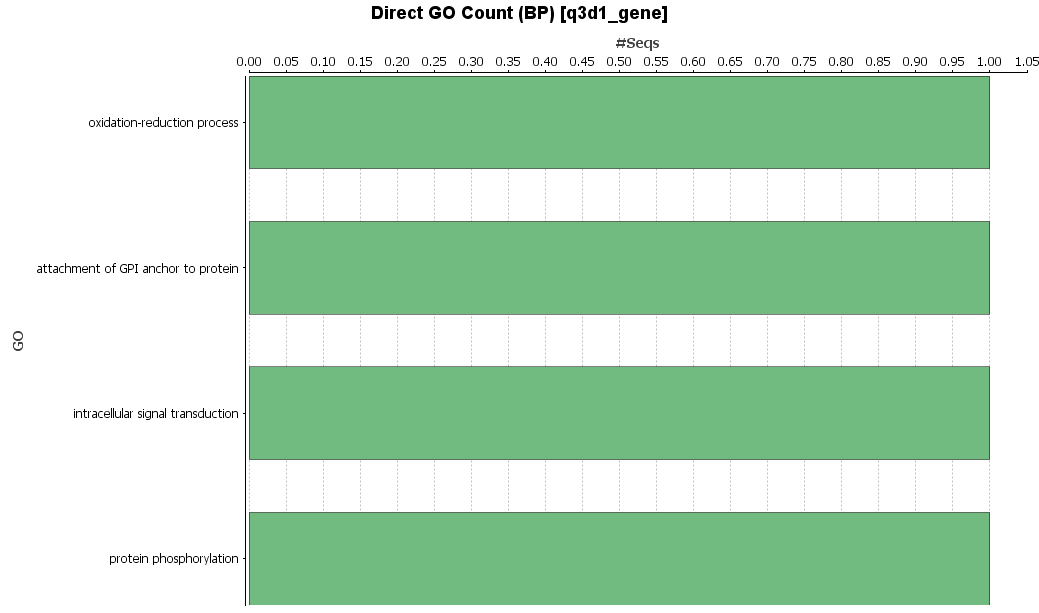


**Fig. S3** Biological process GO category for the genes within the region of QTL Q3D1.


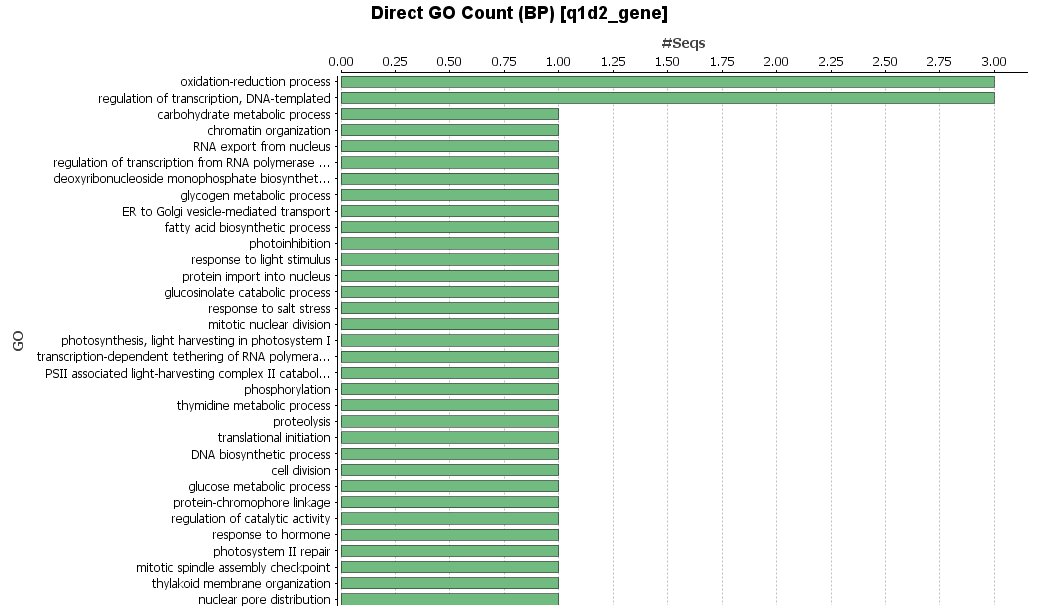


**Fig. S4** Biological process GO category for the genes within the region of QTL Q1D2.


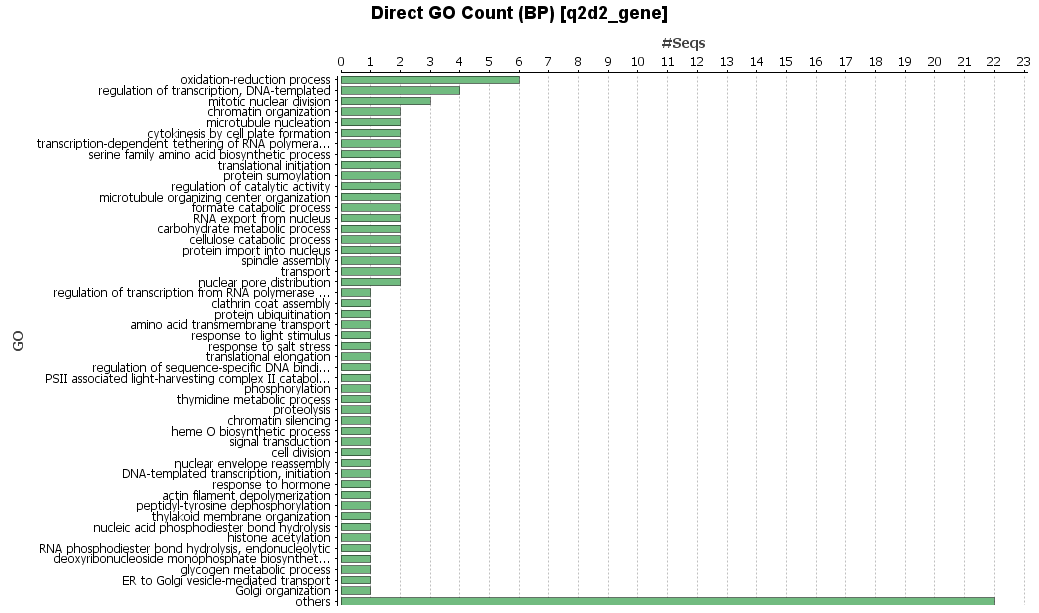


**Fig. S5** Biological process GO category for the genes within the region of QTL Q2D2.


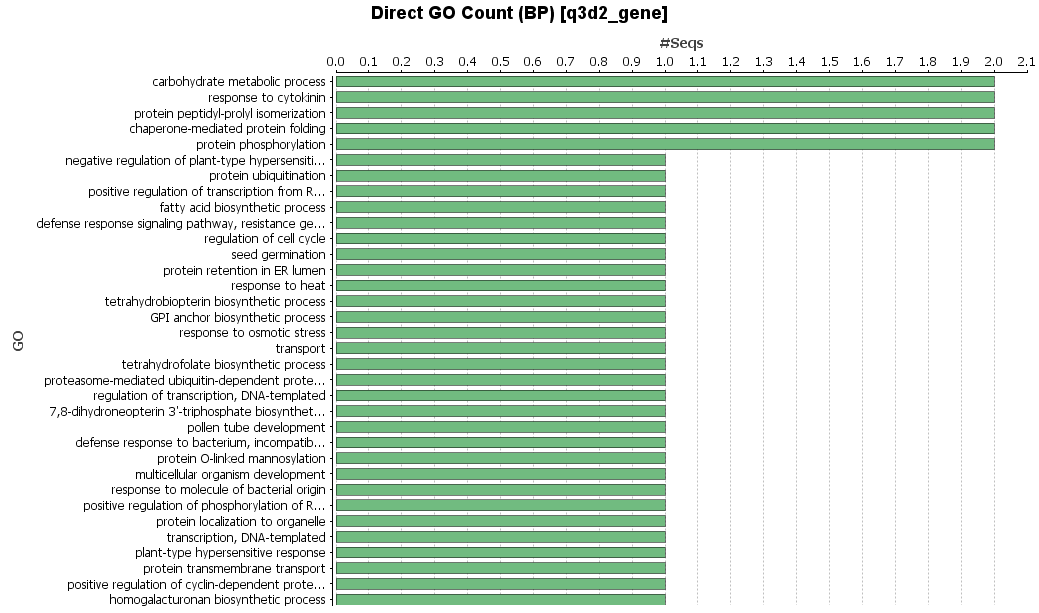


**Fig. S6** Biological process GO category for the genes within the region of QTL Q3D2.


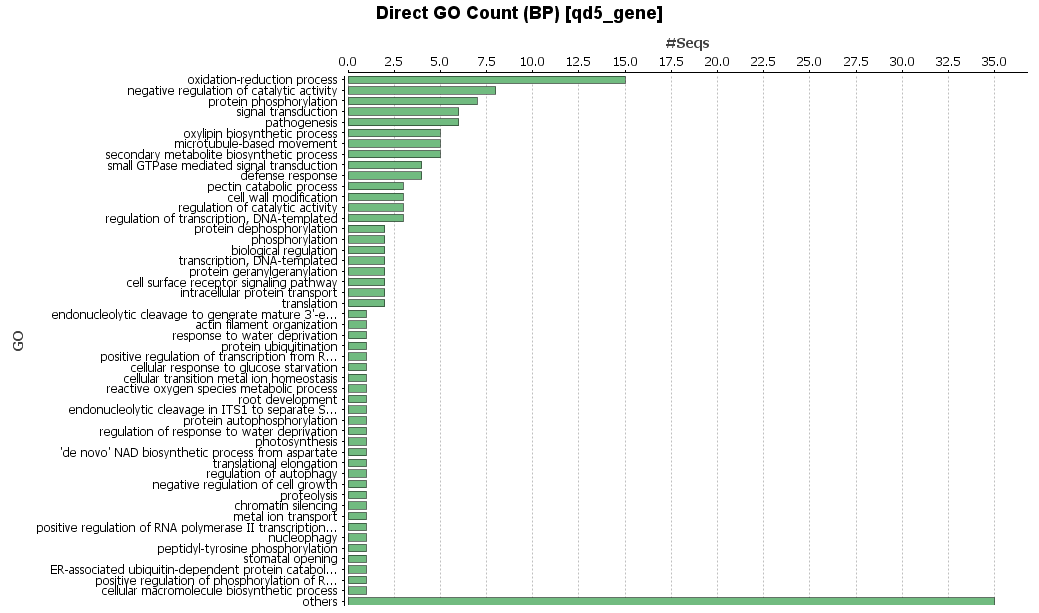


**Fig. S7** Biological process GO category for the genes within the region of QTL QD5.


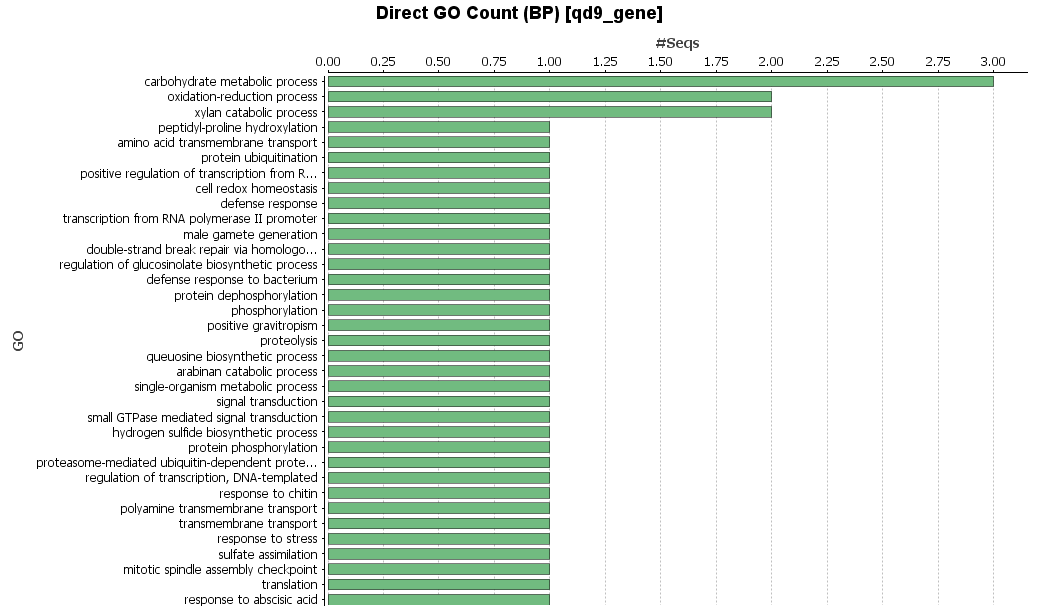


**Fig. S8** Biological process GO category for the genes within the region of QTL QD9.


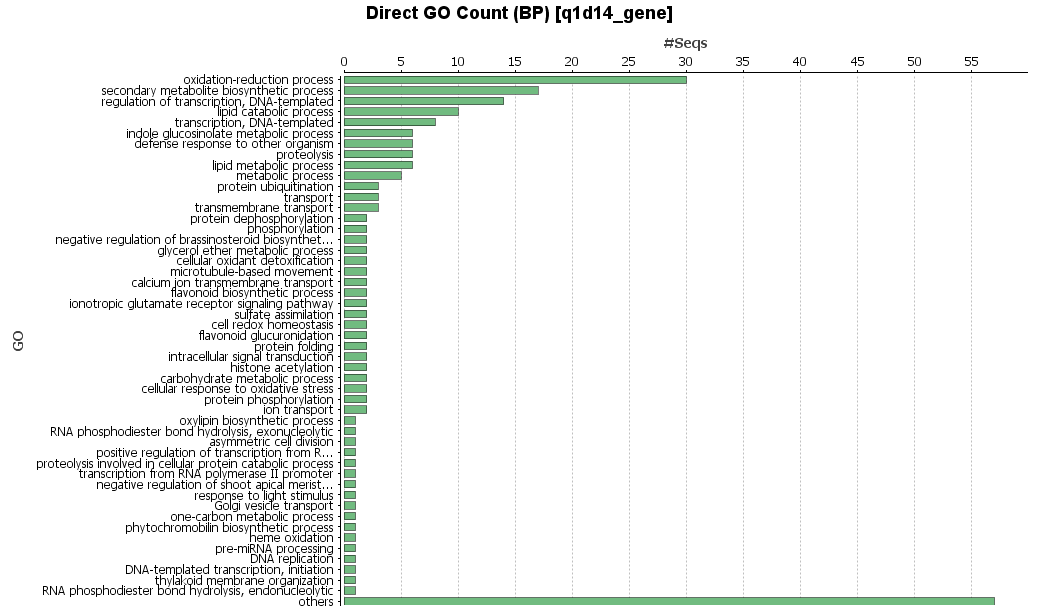


**Fig. S9** Biological process GO category for the genes within the region of QTL Q1D14.


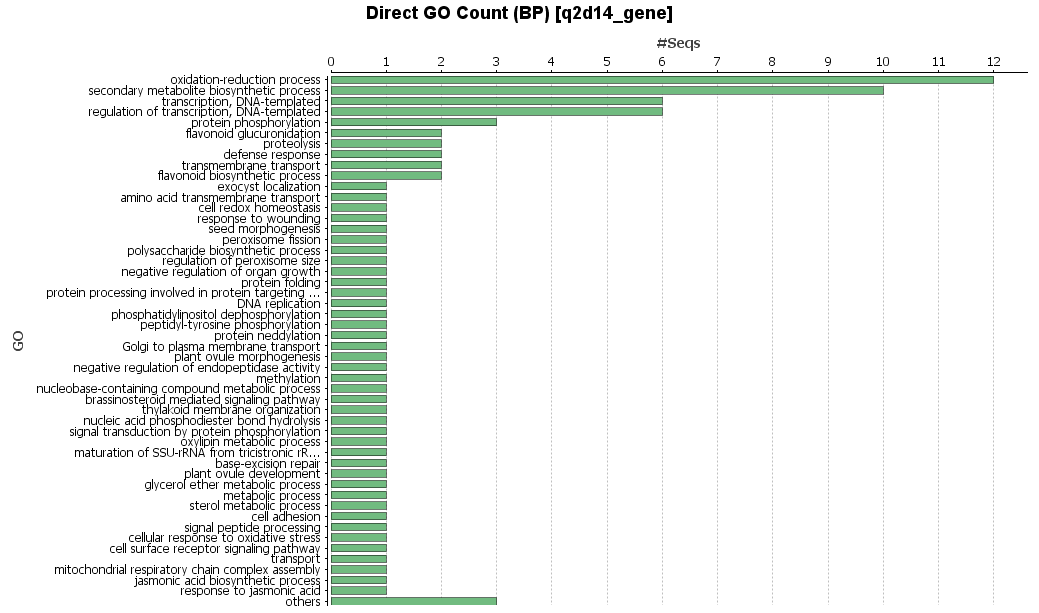


**Fig. S10** Biological process GO category for the genes within the region of QTL Q2D14.


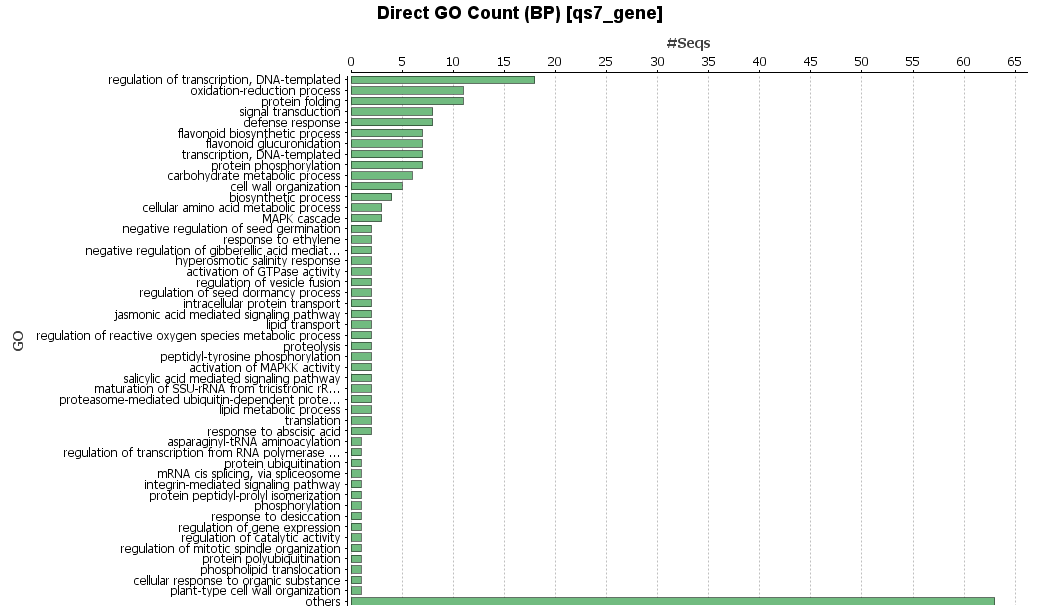


**Fig. S11** Biological process GO category for the genes within the region of QTL QS7.


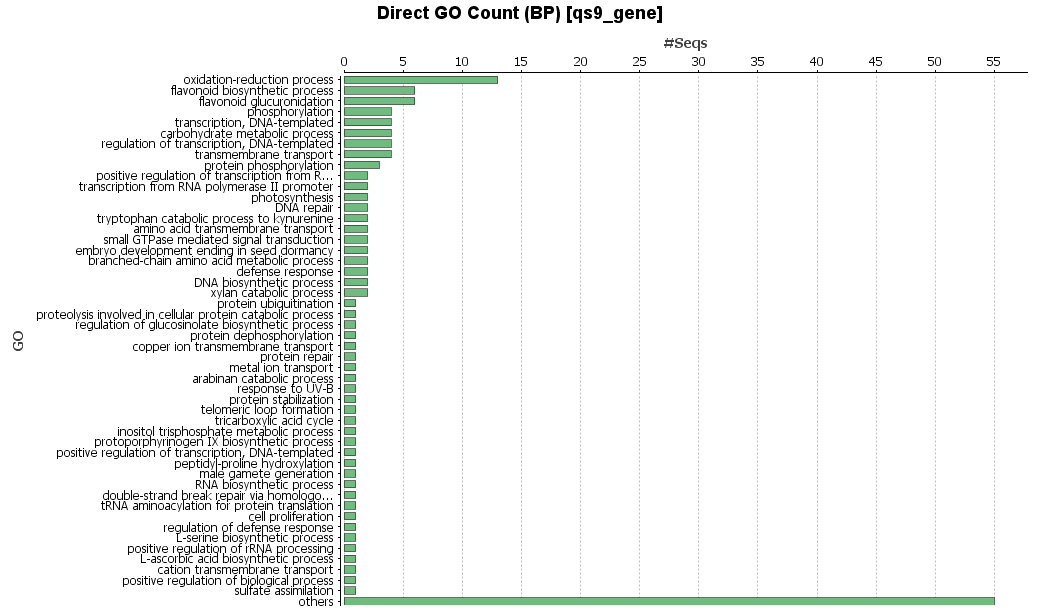


**Fig. S12** Biological process GO category for the genes within the region of QTL QS9.
