# Supplementary material for: MVQTLCIM: composite interval mapping of multivariate traits in a hybrid F1 population of outbred species
Source: BMC Bioinformatics. 2017 Nov 23;18:515. doi: 10.1186/s12859-017-1908-1 (PMC5701343; doi:10.1186/s12859-017-1908-1)
Supplement: Supplementary file 5 — Richards’ growth curves of the 12 QTLs underlying the tree height of Populus, fitted with their genotype values (dot) over time estimated from the multivariate CIM method. The red is for the genotype QQ and the blue for Qq. (PDF 375 kb) [file 12859_2017_1908_MOESM5_ESM.pdf]

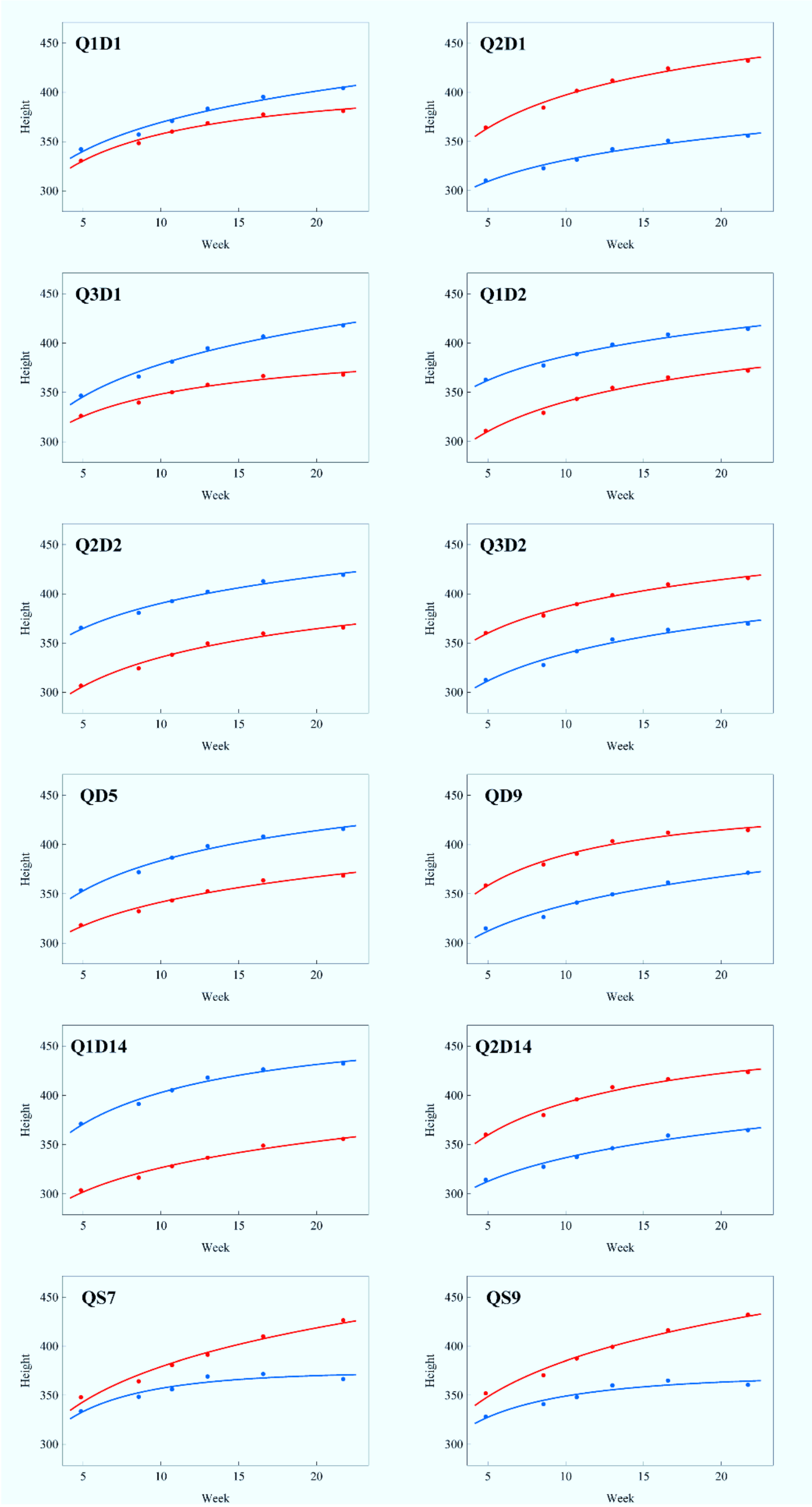

Fig. S13 Richards' growth curves of the 12 QTLs underlying the tree height of *Populus*, fitted with their genotype values (dot) over time estimated from the multivariate CIM method. The red is for the genotype *QQ* and the blue for *Qq*.
