# Supplementary material for: MVQTLCIM: composite interval mapping of multivariate traits in a hybrid F1 population of outbred species
Source: BMC Bioinformatics. 2017 Nov 23;18:515. doi: 10.1186/s12859-017-1908-1 (PMC5701343; doi:10.1186/s12859-017-1908-1)
Supplement: Supplementary file 6 — Plots of the mean cross-validated error against the log of parameter lambda for the female (a) and male (b) SNP datasets. Table S8. SNPs identified to be associated with Populus height by the LASSO method using the two SNP datasets from each parental linkage map. (DOCX 61 kb) [file 12859_2017_1908_MOESM6_ESM.docx]

**LASSO QTL analysis of the two SNP datasets on the female *P. deltoides* and male *P. simonii* linkage maps**

In order to compare with the result of our CIM method implemented in outbred population, we performed QTL analysis for the two SNP data sets on each parental linkage map using the least absolute shrinkage and selection operator (LASSO) approach with the glmnet/R package by choosing the "mgaussian" option (v2.0-10, <http://www.stanford.edu/~hastie/Papers/glmnet.pdf>). To select a stable value of the optimal tuning parameter$\lambda$, the leave-one-out cross-validation was conducted for each dataset, resulting in $\lambda_{\min}=29.90$ and $30.50$ for the female and male dataset, respectively (Figure S14). As a result, a total of 12 SNPs were identified to be associated with the tree height, exactly half of which comes from each SNP dataset. Detailed information of these SNPs is summarized in Table S8. Among these associated SNPs, three were detected consistently by both CIM and LASSO.

1. (b)

**Figure S14** Plots of the mean cross-validated error against the log of parameter lambda for the female (a) and male (b) SNP datasets.

**Table S8** SNPs identified to be associated with *Populus* height by the LASSO method using the two SNP datasets from each parental linkage map

| Map | Linkage  Group | SNP^a^ | Position^b^ (Mb) | T1^c^ | T2 | T3 | T4 | T5 | T6 | Wilks | p-value | Average Heritability (%) | QTL^d^  (Position) |
| --- | --- | --- | --- | --- | --- | --- | --- | --- | --- | --- | --- | --- | --- |
| *P*. *deltoides* | 1 | D1M169 | 36.37 | -15.82 | -15.57 | -16.01 | -17.17 | -17.56 | -18.55 | 0.9078 | 0.0129^*^ | 5.43 | Q3D1 (35.01) |
|  | 4 | D4M63 | 19.74 | -11.09 | -9.45 | -10.89 | -11.15 | -11.18 | -12.38 | 0.9093 | 0.0145^*^ | 2.35 |  |
|  | 5 | D5M3 | 2.24 | -14.46 | -16.18 | -17.80 | -18.58 | -17.64 | -18.57 | 0.8982 | 0.0064^**^ | 5.73 | QD5 (2.24) |
|  | 7 | D7M38 | 9.79 | -9.44 | -8.49 | -9.17 | -9.35 | -10.40 | -11.11 | 0.9449 | 0.1495 | 1.81 |  |
|  | 9 | D9M30 | 6.84 | 15.30 | 18.95 | 20.30 | 21.48 | 21.59 | 20.94 | 0.8675 | 0.0006^**^ | 7.59 | QD9 (8.18) |
|  | 14 | D14M59 | 10.40 | -13.81 | -14.72 | -15.00 | -15.93 | -15.94 | -15.66 | 0.9327 | 0.0707 | 4.44 |  |
|  |  |  |  |  |  |  |  |  |  |  |  |  |  |
| *P*. *simonii* | 1 | S1M10 | 4.57 | 10.30 | 13.32 | 14.52 | 16.58 | 17.44 | 18.89 | 0.8824 | 0.0019^**^ | 4.58 |  |
|  | 5 | S5M20 | 6.51 | 10.98 | 11.18 | 12.31 | 13.33 | 13.20 | 13.10 | 0.9594 | 0.3283 | 2.95 |  |
|  | 6 | S6M5 | 1.50 | 9.98 | 9.94 | 11.99 | 12.49 | 14.09 | 15.25 | 0.9078 | 0.0130^*^ | 2.98 |  |
|  | 10 | S10M4 | 3.81 | 11.10 | 12.08 | 12.87 | 13.07 | 13.74 | 14.59 | 0.9448 | 0.1481 | 3.23 |  |
|  | 12 | S12M34 | 14.35 | 10.87 | 11.64 | 12.62 | 13.03 | 14.38 | 15.33 | 0.9301 | 0.0597 | 3.28 |  |
|  | 17 | S17M24 | 12.41 | 14.30 | 15.20 | 15.76 | 16.97 | 17.67 | 18.02 | 0.9321 | 0.0680 | 5.15 |  |

^a^The SNP name denotes as ‘D/S*M*’, where the first ‘*’ is the number of a linkage group and the second the number of an SNP within a linkage group; ^b^The reference genome of *Populus trichocarpa* v3.0; ^c^T1-T6 are the effect estimates of an SNP over six time points by multivariate regression on the SNPs selected by LASSO; ^d^ The QTL detected by CIM with its positon in bracket is less than 1.5 Mb apart from the SNP.
